# Stochastic decisions support optimal foraging of volatile environments, and are disrupted by anxiety

**DOI:** 10.3758/s13415-024-01256-y

**Published:** 2025-01-09

**Authors:** Alex Lloyd, Ryan McKay, Nicholas Furl

**Affiliations:** 1 Clinical, Educational and Health Psychology, Psychology and Language Sciences, University College London, 26 Bedford Way, London, WC1H 0AP UK; 2 Department of Psychology, Royal Holloway, University of London, London, UK

**Keywords:** Adolescence, Volatility, Stochasticity, Patch foraging

## Abstract

Adolescence is a developmental period of relative volatility, where the individual experiences significant changes to their physical and social environment. The ability to adapt to the volatility of one’s surroundings is an important cognitive ability, particularly while foraging, a near-ubiquitous behaviour across the animal kingdom. As adolescents experience more volatility in their surroundings, we predicted that this age group would be more adept than adults at using exploration to adjust to volatility. We employed a foraging task with a well-validated computational model to characterise the mechanisms of exploration in volatile environments, preregistering the hypothesis that adolescents (aged 16–17; *N* = 91) would exhibit more optimal adaptation of their learning rate to changes in environmental volatility compared with adults (aged 24+; *N* = 90). However, surprisingly, both adolescents and adults exhibited suboptimal adjustment of their learning rate to environmental volatility. In contrast to the learning rate, it was instead participants’ *stochasticity* (i.e., decision variability) that better resembled the adjustment to volatility made by the optimal RL agent. Although heightened stochasticity in the volatile environment led participants to more often trial different responses that facilitated discovery of changes to the environment, we also found that anxiety impaired this adaptive ability. The finding of heightened stochasticity in volatile environments contradicts expectations that the learning rate is responsible for successful adaptation and motivates future work on the deleterious role that anxiety plays when adolescents manage periods of transition.

**Supplementary Information:**

The online version contains supplementary material available at 10.3758/s13415-024-01256-y.

## Introduction

Adolescence (ages 10–24 yr; Sawyer et al., 2018) is a period of profound social and physical change. This key developmental phase encompasses a range of changes to peer and familial relationships (e.g., increasing independence from caregivers; Blakemore & Mills, 2014; Ellis et al., 2012), as well as significant lifestyle changes, such as educational milestones (Eccles et al., 1993). The ability to adapt to such changes is crucial, and theoretical work has suggested that adolescents may be particularly adept at negotiating changeable environments compared with other age groups (Crone & Dahl, 2012; Romer et al., 2017). However, there is still limited empirical evidence to support this claim. The first goal of this study was to examine the computational mechanisms responsible for adjusting to environmental volatility in adolescence and adulthood. Furthermore, the ability to adapt these cognitive mechanisms to one’s surroundings might be threatened by anxiety (Browning et al., 2015), which may be particularly pronounced during adolescence where we see the onset of the majority of anxiety disorders (Lloyd et al., 2023; Rapee et al., 2019). Therefore, our second aim goal to examine the effects of anxiety on the ability to adjust the learning rate to the prevailing environmental volatility.

To accomplish these goals, we leveraged a prominent paradigm from the decision-making literature: patch foraging. Patch foraging tasks are particularly well suited to testing hypotheses about the adaptive features of adolescents’ cognitive flexibility (Duell & Steinberg, 2021), because foraging tasks are reminiscent of the conditions under which these cognitive abilities evolved (Lloyd et al., 2023). During patch foraging, the individual chooses between collecting rewards from discrete patches that deplete the longer the forager remains with the patch and exploring novel patches with fresh distributions of rewards (Charnov, 1976). Empirical work has demonstrated that optimising exploration behaviour while foraging requires the decision-maker to use the rewards gained from previous explore/exploit decisions (i.e., the reward feedback) to determine the point at which to leave the current patch and explore a novel one (Constantino & Daw, 2015; Kolling & Akam, 2017). It has been demonstrated that adolescents explore more optimally than adults while foraging in stable environments and that both adolescents and adults adjust their exploration choices to environments with differing reward availability (Lloyd et al., 2021). However, despite characterisations of adolescence as a period of volatile changes to one’s social and physical environment (Dahl et al., 2018; Ellis et al., 2012; Xia et al., 2021), there is a paucity of evidence about whether adolescents have an improved ability to adjust their exploration between conditions of stability and volatility compared to adults.

A further advantage of patch foraging paradigms is that reward-related decisions can be formally modelled within a reinforcement learning (RL) framework adapted from Marginal Value Theorem (MVT; Constantino & Daw, 2015; Kolling & Akam, 2017). This approach affords building of computational theory to explain how humans adapt their foraging behaviour between stable and volatile environments, including any hypothetically marked ability that adolescents might possess to make this adaptation. Using these RL models, we can directly investigate a computational mechanism known as the “learning rate,” which measures how much recent versus more historic reward feedback guides decision-making. Higher learning rate values denote that the individual makes decisions based on more recent reward feedback (Sutton & Barto, 1998). In stable environments, response options (in the patch foraging paradigm, whether to explore or exploit) and the reward outcomes associated with those options have relationships (known as stimulus-outcome contingencies) that are consistent over time (Behrens et al., 2007). Under these stable conditions, one should not place too much emphasis on recent feedback (i.e., the decision-maker should utilise a lower learning rate), because this can lead the individual to change their expectations about stimulus-outcome contingencies based on random events that are unlikely to reflect the state of the environment (Lawson et al., 2017). Indeed, overweighting recent feedback in stable environments is thought to be an important factor in the development and maintenance of psychological symptoms, such as delusions in schizophrenia (Deserno et al., 2020; Furl et al., 2022). A recent study has demonstrated how the learning rate can be impacted by experiences associated with psychopathology, such as childhood trauma (Lloyd et al., 2022). Conversely, if the association between stimulus-outcome contingencies is known to be rapidly changing (i.e., the association is volatile), unexpected occurrences may signal meaningful changes to the state of the environment. Therefore, when the environment is volatile, using more recent occurrences as a basis for one’s decisions (i.e., utilising a higher learning rate) is beneficial (Browning et al., 2015). Adjusting one’s behaviour to this contrast between stability and volatility in the environment is important to successfully reap rewards from one’s surroundings (Behrens et al., 2007).

Examining how humans negotiate volatile and stable environments is important to consider, because foraging tasks reflect key computations needed across a breadth of real-world contexts relevant to adolescents and adults (Lloyd et al., 2023). One such computation is the estimation of the *background reward rate*, otherwise known as the richness of the environment (Gabay & Apps, 2021) and refers to the average rewards expected from the environment for each unit of time (Charnov, 1976). The background reward rate is key to solving sequential decision-making problems, such as choosing a romantic partner or accepting a job opportunity (Constantino & Daw, 2015; van de Wouw et al., 2022). Indeed, adjusting decision-making to the relative volatility of the background reward rate has important implications for real-world decision-making. We define volatility as a condition in which reward contingencies in the environment switch periodically (Piray & Daw, 2021). Volatile conditions reflect a breadth of decision-making contexts. For example, the decision to accept a mortgage offer may rely on estimating the relative value of other offers on the market and how stable the quality of those options are likely to be. In conditions where the quality of such options is volatile (e.g., during the 2008 financial crisis), an optimal decision-maker may rely on immediate changes to the quality of mortgage offers to determine whether to stick with their current offer or explore others on the market.

Adolescents appear to utilise learning rates more appropriate to the relative volatility of the environment compared with adults. For example, in volatile environments, adolescents more optimally weight recent evidence and accrue more rewards compared with other children and adults (Eckstein et al., 2020). Some evidence suggests that adolescents also perform more optimally than adults in stable environments. Davidow and colleagues (2016) found that adolescents, because they utilised a lower learning rate than adults in a stable environment, could better learn the stimulus-outcome contingencies, more often select the stimulus that led to a reward and collect more points. However, a contrasting body of literature has found that adults more optimally set their learning rates to stable environments relative to adolescents (Christakou et al., 2013; Master et al., 2020; Nussenbaum et al., 2022). Therefore, there is mixed evidence regarding whether adolescents or adults more optimally tune their learning rates in stable conditions. Nevertheless, the limited developmental studies that have compared adolescents and adults in volatile conditions suggest that adolescents more optimally set their learning rate to these unstable environments (Eckstein et al., 2020). Indeed, adolescents’ improved ability to navigate volatile conditions would be consistent with the characterisation of this developmental period as a time of increased psychosocial change (Blakemore, 2018; Spear, 2000). We therefore suggest that when presented with environments that change from stable to volatile, adolescents should be more adept at negotiating these changing stimulus-outcome contingencies, whereas adults will be less adept as their learning rates will be set to optimally navigate stable environments.

These studies do not, however, mean that adults are unable to adjust their learning rate to the prevailing volatility of the environment, as this ability is well-established in adults (Behrens et al., 2007; Piray & Daw, 2020). What is not yet known is if adolescents are more adept than adults at adjusting their learning rate within the same experiment. Testing this question can also provide novel insight into whether serial tasks with exploration decisions (e.g., foraging tasks) rely on the same computational mechanisms (i.e., learning rate) as RL tasks with simultaneous options, such as n-armed bandit tasks (Eckstein et al., 2020). If not, then the learning rate may not control adaptation to environmental volatility in serial decision tasks, and perhaps alternative computational mechanisms instead mediate explore/exploit choices. We experimentally manipulated the prevailing volatility of a serial decision-making task to examine whether the learning rate is the operative computational mechanism responsible for adjusting decision-making to the statistics of the environment as in previous research (Browning et al., 2015) or whether another computational mechanism is responsible for this cognitive ability.

To empirically test how human participants on serial decision tasks adjust their explore/exploit choices between conditions of stability and volatility, along with whether adolescents exhibit greater cognitive flexibility relative to adults, we manipulated the volatility of the environment in a foraging paradigm. We also simulated RL models to estimate the optimal weight participants should place on recent versus historic feedback (i.e., the optimal learning rate) in these environments, with these simulations confirming that it was optimal to utilise a higher learning rate in the volatile environment relative to the stable environment. On the expectation that participants will utilise a higher learning rate in the volatile environment relative to the stable environment, our first pre-registered hypothesis (link: https://osf.io/p3md9/?view_only=11acb14fab5e4141861670a0fd81714b) was that adults will exhibit a smaller difference in learning rate between the two environments whereas adolescents will exhibit a larger difference, consistent with the simulated optimal RL agent.

The RL model we used also contains a stochasticity parameter, which measures the degree to which participants randomly deviate from the option that, to their current knowledge, has the highest expected value. Stochastic decision-making can also promote exploration, leading the individual to identify changes to their environment faster than a decision-making strategy that relies on exploiting the option with the highest expected value (Wilson et al., 2021), and may therefore contribute to the ability to adjust explore/exploit decision-making to the prevailing environmental volatility (Gershman, 2018). As such, we report exploratory analyses of this model parameter to examine whether stochasticity might also provide a computational mechanism for adapting to volatility.

The ability to optimally adjust decision-making to environmental volatility is disrupted by psychopathology, specifically anxiety. Anxiety disorders can lead to poorer instrumental learning (Alvares et al., 2014) and a poorer ability for adults to adapt their learning rate between stable and volatile environments (Browning et al., 2015). As such, we expect anxiety to disrupt adolescents’ and adults’ ability to adjust their explore/exploit choices between stable and volatile environments. While other psychopathologies have been associated with biased explore/exploit decision-making (e.g., depression and psychosis; Lloyd et al., 2024), anxiety has specifically been demonstrated to disrupt adults’ ability to adapt between stable and volatile environments (Browning et al., 2015). Indeed, the computational mechanisms that are biased in individuals with anxiety may be informative in understanding the presentation of these disorders, such as behavioural inflexibility (Figueiredo et al., 2023). Considering the role of anxiety in foraging tasks specifically is important given the prevalence of foraging choices across human decision-making (Lloyd et al., 2023). To date, there is relatively limited understanding of how anxiety relates to foraging decisions compared with other contexts (e.g., n-armed bandit tasks). For example, understanding how anxiety affects foraging choices may help to understand difficulties adolescents have in adjusting to novel environments after significant periods of transition, such as beginning a university programme. Our second preregistered hypothesis, therefore, was that across age groups, higher rates of anxiety would be associated with a less optimal adjustment of participants’ learning rate between the stable and volatile environments.

Finally, our third preregistered hypothesis was that we would replicate previous research (Lloyd et al., 2021), demonstrating that adolescents explore more than adults in stable foraging environments, which we measured in the current study as the point at which participants leave their current patch to explore a novel one (i.e., their leaving threshold).

## Methods

### Participants

We recruited 181 participants including 91 adolescents (M_age_ = 16.32, SD_age_ = 0.49, 70.33% female) and 90 adults (M_age_ = 33.54, SD_age_ = 7.12, 68.09% female). The sample size was based on a priori power calculations using G*Power (Faul et al., 2007). For our first hypothesis, we conducted an *a priori* power analysis for an interaction effect with one between- and one within-participant factor in an ANOVA (alpha = 0.05, power = 0.8, Cohen’s f = 0.25), with the effect size based on previous research using this paradigm (Lloyd et al., 2021). For our second hypothesis, we conducted an a priori power analysis for the covariate effect within a general linear model (alpha = 0.05, power = 0.8, effect size f^2^ = 0.15). For our third hypothesis, we conducted an *a priori* power analysis for an independent *t*-test (alpha = 0.05, power = 0.80, effect size *d* = 0.5), with the effect size based on our previous research (Lloyd et al., 2021). These three power analyses indicated that we required a minimum of 160 participants to test our hypotheses. Adolescent participants were recruited in Surrey, UK. Adult participants were recruited through Prolific (www.prolific.co), and we restricted the location of participants recruited through this platform to the UK to match the location of the adolescent sample. Descriptive statistics of demographic variables for the adolescent and adult groups can be found in Supplementary Table 2.

The age of the adolescent sample was determined based on evidence that novelty-seeking peaks during middle-adolescence (approximately aged 16–17 yr; Steinberg et al., 2018), which allowed us to examine hypotheses about the potential adaptive role of exploration choices during adolescence. A further reason motivating the recruitment of this age range was based on evidence that value-guided memory evidence increases across development (Nussenbaum et al., 2020, 2021). This is important in the context of our modelling approach, because it is not always possible to disentangle whether the stochasticity parameter reflects an explicit strategy used to learn about the individual’s surroundings, or unguided randomness that may be associated with a poorer ability to learn (Zorowitz et al., 2023). The development of value-guided memory may therefore support older adolescents to use more complex strategies to navigate foraging environments, such as the deliberate use of stochastic decision-making. We restricted our adult sample to a minimum age of 24, because this is when regions implicated in foraging choices are fully matured (Sawyer et al., 2018; Tervo-Clemmens et al., 2023). Further, the upper age limit was set at 50 yr, because there is evidence that exploration while foraging declines into older adulthood (Mata et al., 2013). Because there was an uneven distribution of ages within our adult sample, we opted to treat age as a discrete variable in the analyses rather than a continuous variable, as participants older than age 40 yr were underrepresented in this sample (see Supplementary Materials for age distributions in the adult sample).

### Procedure

Participants completed the study online during the COVID-19 pandemic. Both adolescent and adult samples were recruited within a 16-day window to avoid systematic differences between the samples that could arise from population level changes in anxiety associated with the pandemic (Fancourt et al., 2021). We note that hypotheses were preregistered after data collection had begun, but prior to viewing any of the data. The study was hosted on Gorilla.sc, an online platform for behavioural research (see https://app.gorilla.sc/openmaterials/863981 for a copy of the task). Adolescent participants were provided a link to complete the task in a school session supervised by their class teacher and adults were directed automatically to the task through Prolific.

**Fig. 1:**
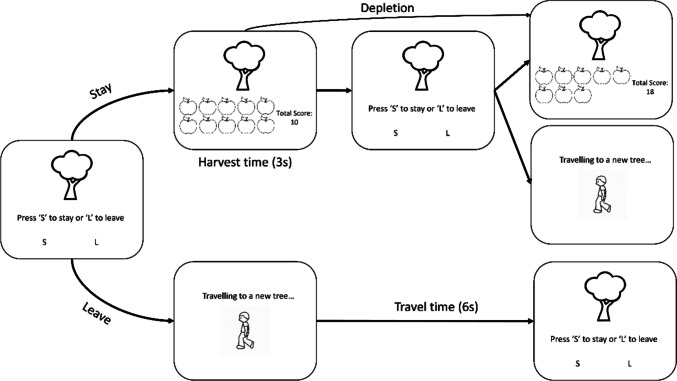
Patch foraging task. The leftmost panel highlights the option to stay or leave, which is presented to participants when they enter the environment. If participants choose to stay with the patch, they see the number of rewards collected on that trial, along with their cumulative score (topmost panel), after which they are presented again with the option to stay or leave. Alternatively, if a participant’s choice on the leftmost panel is to leave and explore a new patch, they are presented with a travel screen for 6 s before arriving at a new patch (bottommost panels). In the volatile environment, the number of apples harvested on the first stay decision and the rate at which apples depleted within patches changed every 30 trials, whereas in the stable environment these properties remained constant throughout the block (i.e., foraging environment)

Once participants began the study, they were provided with a consent form detailing the study protocol and the contact details of the researchers. After providing consent, participants completed a demographic questionnaire and the foraging task. We utilised an apple patch foraging paradigm (Constantino & Daw, 2015) to examine explore/exploit decision-making in conditions of stability and volatility (Fig. 1). Participants attempted to collect as many rewards as possible within a fixed time limit of seven minutes, which was outlined explicitly to participants before they began the task. This time limit was based on previous studies (Constantino & Daw, 2015; Lloyd et al., 2021), as well as simulation data to ensure participants encountered both rich and poor portions of the volatile environment. Rewards were operationalised as apples, patches were operationalised as trees that were available for foraging, and environments were described as “orchards” from which participants collected apples. If participants chose to stay in the current patch and exploit it, they would be presented with the number of apples collected on that trial alongside their cumulative number of apples collected. Rewards would deplete on each trial until zero apples remained in the patch, which participants could continue to exploit although they would not collect any further rewards. On each trial, participants were also provided with the option to leave and explore a novel tree, which would have a fresh distribution of rewards. Explore decisions incurred a time cost during which no new rewards could be harvested, which was fixed at six seconds in both the stable and volatile environments. As participants had a fixed amount of time to collect rewards, exploring new patches limited the time available for participants to harvest apples. Participants had up to two seconds to decide whether to explore or exploit on each trial. If they did not make a response within this window, they would be presented with a timeout screen after which they arrived at a new patch. Timeout trials incurred a longer wait time than exploring to ensure that there was no benefit from not responding within the allocated time. Trials where participants timed out were excluded from the analyses (see Measures below).

Before completing the main task, participants completed a practice foraging environment. The practice environment lasted for two minutes, and participants were instructed that rewards collected during this practice would not count towards their overall score. In this practice environment, the initial number of rewards on patches was drawn from a Gaussian distribution with a mean of 10 (standard deviation [SD] = 1), and the depletion rate was drawn from a Gaussian distribution with a mean of 0.90 (SD = 0.07). Once the practice task was complete, participants were provided with a reminder of key information about the task. Specifically, participants were reminded that the aim of the task was to collect as many apples as possible and that they would have seven minutes in each “orchard” or foraging environment. Moreover, participants were instructed that the environments might differ from one another, which may affect whether they should stay for more or less time with patches. However, we did not provide explicit information about how the environments would differ as a key feature of foraging tasks is that participants should independently learn to adapt their explore/exploit choices to the reward statistics of the environment (Gabay & Apps, 2021). In the stable environment used in the main task, the distribution of rewards on new patches (i.e., their initial richness) was set at a mean of 10 (SD = 1). Rewards in patches underwent exponential depletion, in which the current harvest was multiplied by a value drawn from a Gaussian distribution with a mean of 0.88 (SD = 0.07).

In the volatile condition of the main task, the initial richness of rewards on new patches and depletion rate within patches switched after every 30 trials (i.e., exploit or explore choices) to create rich and poor portions of this environment. The change in reward statistics occurred even when the participant was part-way through exploiting a patch. This design choice was made to ensure that participants did not assign changes in the reward statistics to individual patches and instead assign variability to the foraging environment. Further, participants were not explicitly informed when the volatile environment shifted between rich and poor portions, though they were informed when they moved to a different “orchard” (i.e., from the stable to the volatile environment, or vice versa). In the rich portion of the volatile environment, the mean initial richness of patches was 13 apples (SD = 1) with an exponential depletion rate drawn from a Gaussian distribution with a mean of 0.94 (SD = 0.07). In the poor portion of the volatile environment, the mean initial richness of patches was seven (SD = 1), and the exponential depletion rate was drawn from a Gaussian distribution with a mean of 0.76 (SD = 0.07). We did not manipulate the time cost of exploration as in previous studies (Constantino & Daw, 2015; Lloyd et al., 2022) to avoid participants remaining in the poor-quality portion for substantially longer than the rich quality portion. Based on pilot data we found that using fewer than 30 trials could have allowed participants to experience only a single patch in each portion if they chose to exploit the patch until it was fully depleted. Furthermore, we opted to present the rich and poor portions of the volatile environment as blocks rather than increase the reward variability in the volatile environment due to the focus on volatility (as opposed to environmental stochasticity; see Piray & Daw, 2021). Specifically, widening the reward distribution would mean that the forager still converges on a consistent estimate of the background rate. In contrast, our blocked design required participants to adjust their estimate of the background reward rate between rich and poor portions, adapting their explore/explore choices accordingly.

The sequence in which the stable and volatile environments were presented was counterbalanced across participants, as was the presentation of the rich and poor portions in the volatile environment. The foraging task took approximately 16 min to complete in total. After completing the main task, participants completed the GAD-7 questionnaire and, for adolescent participants only, the Pubertal Scale. Finally, participants were told their final score and debriefed as to the aims of the study. All participants were compensated a minimum of £3 for their participation. In addition to this base payment, participants were awarded a performance-dependent bonus which was worth up to £2 and was calculated as a proportion (0.12%) of their total score across both environments.

### Measures

#### Leaving thresholds

For our behavioural analyses (see pre-registered Hypothesis 3), we were interested in participants’ exploration behaviour, operationalised in the present study as their leaving thresholds on the foraging task. We assume as in previous research (Constantino & Daw, 2015) that participants select a value of rewards as their “threshold” and that when they expect the number of rewards from the next exploit decision to fall below this threshold value, they leave to explore a new patch. This variable was calculated as a behavioural measure that did not account for how participants learned the background reward rate and was therefore independent of model-based analyses, which we adopted based on previous research (Constantino & Daw, 2015). We calculated this variable to identify whether participants adjust their exploration choices in response to the manipulations we made between the stable and volatile environments, which is important in the context of the present study to identify whether participants adjusted their decision-making between rich and poor portions of our novel volatile foraging environment. These behavioural findings were used as a basis to identify whether there were any empirical observations in the data that modelling could be used to explain.

Higher values on the leaving threshold indicate participants explore more frequently. We calculated participants’ leaving threshold as the average number of rewards harvested from the previous two exploit decisions before they chose to explore (Constantino & Daw, 2015). We compared participants’ leaving threshold in the stable environment to the optimal leaving threshold for this environment, which was calculated by conducting a grid search across leaving thresholds ranging from 1–10 in increments of 0.001 and identifying which leaving threshold yielded the highest number of rewards (Lloyd et al., 2021). The maximum of the simulated leaving threshold was set at 10 because thresholds above this value would lead to an irrational exploration-only policy. The minimum of the simulated leaving threshold was one as values below this threshold would indicate the agent assumes the background reward rate to be less than one unit of reward, which is untrue for most foraging environments employed in behavioural research. To calculate the optimal leaving thresholds in the volatile environment, we simulated an agent that used separate leaving thresholds for the rich and poor portions of this environment. The combination of leaving thresholds that yielded the highest number of rewards was considered the optimal combination of leaving thresholds in this environment (see Supplementary Materials for further information).

#### Anxiety

To measure anxiety, we utilised the General Anxiety Disorder-7 scale (GAD-7; Spitzer et al., 2006). The questionnaire asks participants to report how often they have been bothered by a series of problems over the past 2 weeks. For example, participants are asked how often they have been bothered by “Worrying too much about different things?” on a four-point Likert scale, ranging from “Not at all” to “Nearly every day.” Total scores were used. Scores of 5, 10, and 15 reflect mild, moderate, and severe anxiety, respectively (see Supplementary Fig. 7 for the distribution of anxiety scores in adolescents and adults). This scale demonstrated excellent reliability in the sample recruited for this study α = 0.90.

#### Demographic information

Participants were asked to report their age, gender, ethnicity, and highest level of education. We asked adolescent participants to report their parents’ highest level of education, which can be used to approximate socioeconomic status for developmental samples (Steinberg et al., 2018). Descriptive statistics regarding participants’ demographic information are reported in Supplementary Table 2.

#### Pubertal scale

The Pubertal Status scale (Carskadon & Acebo, 1993) measures adolescents’ pubertal development, which can be distinct from their chronological age. The scale comprises five questions, which ask whether developmental milestones, such as physical growth, are underway or complete. Items are scored on a 4-point Likert scale, with higher values indicating the individual is more highly developed. Individuals’ pubertal development is calculated as a mean score on the scale. We note that as the adolescents recruited for this study were aged 16–17 yr, scores on this scale were negatively skewed.

#### Computational measures

Using computational modelling, we derived estimates of the optimal learning rates and decision stochasticity as well as estimates of participants’ learning rates and decision stochasticity in the stable and volatile foraging environments. We then utilised estimates of participants’ parameters as dependent measures in our behavioural analyses, in addition to the leaving threshold measure described above. The computational model we used in the present study was derived from a prominent theory of optimal foraging known as Marginal Value Theorem. This theorem prescribes that the forager should leave their current patch to explore a novel one when the rewards expected from exploiting the current patch fall below the forager’s estimate of the background reward rate, which is inferred from the background reward rate in an environment (Charnov, 1976; Gabay & Apps, 2021). Drawing on the reinforcement learning framework, Constantino and Daw (2015) developed a model that describes how participants learn this average reward rate of the environment by integrating reward-feedback into their estimation of this value:

1 $${p}_{i}={(1-\alpha )}^{{T}_{i}}\frac{{s}_{i}}{{T}_{i}}+\left(1-{\left(1-\alpha \right)}^{{T}_{i}}\right){p}_{i-1}$$

The equation describes how the participant updates their estimate of the average reward rate (*p*) on each trial (_*i*_). The term (*T*_*i*_) denotes the time cost associated with either an explore (6s) or exploit (3s) decision and (*s*_*i*_) denotes the reward (operationalised as the number of apples) received on each trial. The equation additionally contains the free parameter α, which measures the complement of participants’ learning rate (i.e., the learning rate is 1-α). This learning rate value captures the degree to which participants weight recent information in their decision-making (Sutton & Barto, 1998). Participants’ estimation of the average reward rate is then entered into a SoftMax function, which estimates the probability that participants will choose to exploit on each trial:

2 $$P\left({a}_{i}=stay\right)=1/(1+exp \left(-(1 + \beta [{{k}_{i-1}s}_{i-1}-{p}_{i-1}h]\right) ))$$

In Equation (2), higher values on the stochasticity or inverse temperature parameter *β* indicate the participant more reliably selects the option which they believe to have the highest expected value, whereas lower values on *β* indicate the participant more often randomly diverts from the option which they believe to have the highest expected value (also known as exhibiting more stochastic choices). Exhibiting more stochasticity (i.e., utilising lower values of *β*) can be beneficial in environments with volatile stimulus-action contingencies, as it can lead the agent to identify these changes faster than reliably selecting the option the agent believes to yield the highest reward. The term *k*_*i-1*_*s*_*i-1*_ refers to the last known reward obtained from exploiting the patch (*s*_*i*_), multiplied by the depletion rate *k*_*i*_. In the present study, the value of *k* was updated on each trial (_*i*_) and was calculated through averaging the true depletion rate experienced on all previous exploit decisions (as in previous research; Constantino & Daw, 2015). Finally, the term *p*_*i*_*h* refers to participants’ current estimate of the background reward rate (*p*_*i-1*_), multiplied by the harvest time (*h*), which was fixed at three seconds in both environments and was inclusive of the time it took participants to make an explore/exploit decision. This equation therefore incorporates the comparison between the background reward rate as well as the value of the current patch, as specified in MVT (Charnov, 1976). However, the model does not assume participants utilise a static leaving threshold and rather the values that determine whether the participant leaves their current patch (i.e., their leaving threshold) are updated on each trial and entered into a probabilistic equation to determine the likelihood that the participant will stay on any given trial.

Parameters were estimated from participants’ behavioural data individually using maximum likelihood estimation. Specifically, we used the optim function for R (R Core Team, 2021), which implemented a Nelder-Mead algorithm to estimate parameters from participants’ data with bounds for the learning rate parameter {0, 1} and stochasticity parameter {0, 5}. Further details about model validation and comparison are reported in the Supplementary Materials.

We expected that the optimal strategy (i.e., as implemented by an optimal RL agent) would be to increase learning rate in the volatile environment and that participants would also reproduce this strategy. Our first two preregistered hypotheses assert that participants will adjust their learning rates to the prevailing volatility of the environment, with this adjustment being larger and more optimal for adolescents than adults (Hypothesis 1) and reduced and less optimal for more anxious individuals (Hypothesis 2). In exploratory analysis, we tested which values of the stochasticity parameter empirically yielded the highest reward rates in each environment. We tested simulated models with different combinations of the free parameters (α, representing complement of the learning rate; and β, representing stochasticity), which allowed us to identify which parameter values yielded the greatest number of rewards in each environment (see Supplementary Materials). These simulations confirmed that it was optimal to adopt a higher learning rate in the volatile environment relative to the stable environment. In addition, exploratory analyses of these simulations demonstrated that it was optimal to have more stochastic behaviour in the volatile environment relative to the stable environment.

### Design

We describe the design specified in our pre-registration document (https://osf.io/p3md9/?view_only=11acb14fab5e4141861670a0fd81714b), although further exploratory analyses were conducted in addition to the statistical tests described in the preregistration. The preregistered design to test Hypothesis 1 was a 2×2 mixed factorial design with two independent variables: age group (adolescent or adult) and foraging environment (stable or volatile). Our first hypothesis was that participants would increase their learning rate in the volatile condition, relative to the stable condition. Moreover, we predicted that the degree to which adolescents adjusted their learning rate between the stable and volatile environments would be greater than adults. As such, the dependent variable of interest for this first hypothesis was participants’ learning rate. Because we were also interested in the effect of anxiety on participants’ ability to adjust their learning rates between environments (Hypothesis 2), self-reported anxiety was entered into a regression model as a predictor of the difference between participants’ learning rate in the stable and volatile environments. Our third hypothesis was that adolescents would explore more than adults in the stable environment. As such, the dependent variable of interest for the third hypothesis was participants’ leaving thresholds, which measured participants’ rates of exploration. Adopting this group-based approach for our analyses of age effects allowed us to address Hypothesis 3, which was to replicate previous work that utilised a similar design (Lloyd et al., 2021). In addition, exploratory analyses repeated those that were preregistered for the learning rate parameter, but instead inserted the other free parameter in this model, decision stochasticity (β), as an exploratory dependent variable.

## Results

### Behavioural results

**Fig. 2:**
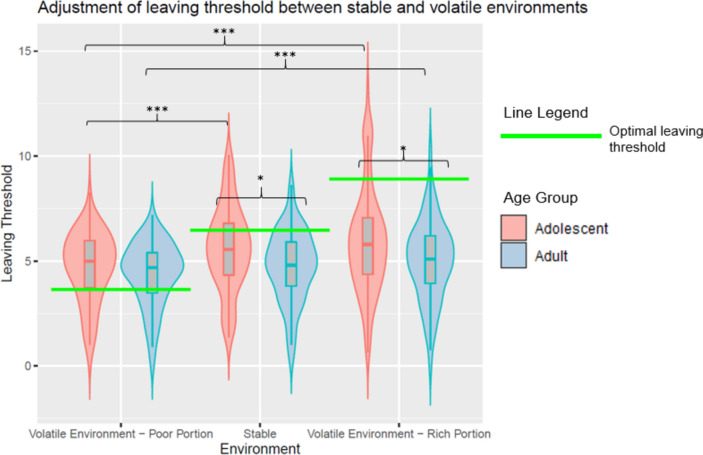
Violin plot demonstrating that, compared with adults, adolescents exhibited greater adjustment of their leaving threshold between rich and poor portions of the volatile environment, and between the volatile and stable environments. Boxplots indicate the median leaving threshold for each potion, along with upper and lower quartiles. Horizontal green lines denote the optimal leaving threshold for that environment, or portion within the environment. *p < .05; **p < .01; ***p < .001

We first report analyses of model-free measures of participants’ exploration choices (i.e., their leaving threshold). Supporting our third preregistered hypothesis, we replicated previous results (Lloyd et al., 2021) that demonstrated that adolescents explored more than adults in the stable environment, measured by the threshold of apples required before they chose to travel to a new patch (i.e., their leaving threshold; *t*(179) = 3.07, *p* = .002, Cohen’s *d* = 0.46; Fig. 2).

We also examined whether participants adjusted their leaving thresholds between the rich and poor portions of the volatile environment, as well as between the volatile and stable environments. According to MVT, an optimal forager should adjust their exploration to the background reward rate of the environment, exploring more when the background reward rate is higher and less when the background reward rate is lower. In this novel volatile foraging task, we manipulated the background reward rate within a single environment such that there were rich and poor portions within the volatile environment. This manipulation is in comparison to previous studies that have manipulated the background reward rate between environments only (Lloyd et al., 2021). To validate this novel behavioural task, we conducted exploratory analyses on participants’ leaving thresholds to examine whether they adjusted their exploration to changes in the background reward rate in the volatile environment. We entered environmental richness (volatile rich, volatile poor, and stable) and age group (adolescent or adult) as factors into a 2*3 repeated measures ANOVA, with leaving threshold as the dependent variable. There was a main effect of environmental richness on participants’ leaving thresholds (Greenhouse-Geisser corrected; *F*_(1.78,307.75)_ = 40.94, *p* < .001, η^2^_*p*_ = 0.19). Participants utilised a higher leaving threshold in the rich portion of the volatile environment compared to the stable environment (*p*_*bonf*_ = .011; corrected for three comparisons) and compared to the poor portion of the volatile environment (*p*_*bonf*_ < .001; Fig. 2). Participants also utilised a higher leaving threshold in the stable environment compared to the poor portion of the volatile environment (*p*_*bonf*_ < .001). Adolescents utilised a higher overall leaving threshold demonstrated through a main effect of age group (*F*_(1,173)_ = 7.48, *p* = .007, η^2^_*p*_ = 0.04), consistent with previous work (Lloyd et al., 2021).

There was also an interaction between age group and environmental richness on the degree to which participants adjusted their leaving thresholds (Greenhouse-Geisser corrected; *F*_(1.78,307.75)_ = 4.30, *p* = .018, η^2^_*p*_ = 0.02). Post hoc tests using the Bonferroni correction for 15 comparisons demonstrated that compared with the poor portion of the volatile environment, adolescents exhibited a significantly higher leaving threshold in the stable environment (*p*_*bonf*_ < .001) and the rich portion of the volatile environment (*p*_*bonf*_ < .001; Fig. 2). In contrast, compared with the poor portion of the volatile environment, adults only exhibited a higher leaving threshold in the rich portion of the volatile environment (*p*_*bonf*_ < .001), but not the stable environment (*p*_*bonf*_ = .170). These findings suggest that adolescents and adults could adjust their leaving threshold to changes in richness within the volatile foraging environment, but only adolescents effectively adjusted their leaving threshold to changes in environmental richness in the volatile environment, as well as between the stable and volatile foraging environments. However, it is unclear whether participants’ adjustment of their leaving threshold in response to changes in the environmental richness is accounted for by their learning rate or stochasticity, a question we turn to in the computational modelling section below.

### Computational modelling results

#### Participants use a lower learning rate in volatile environments

**Fig. 3:**
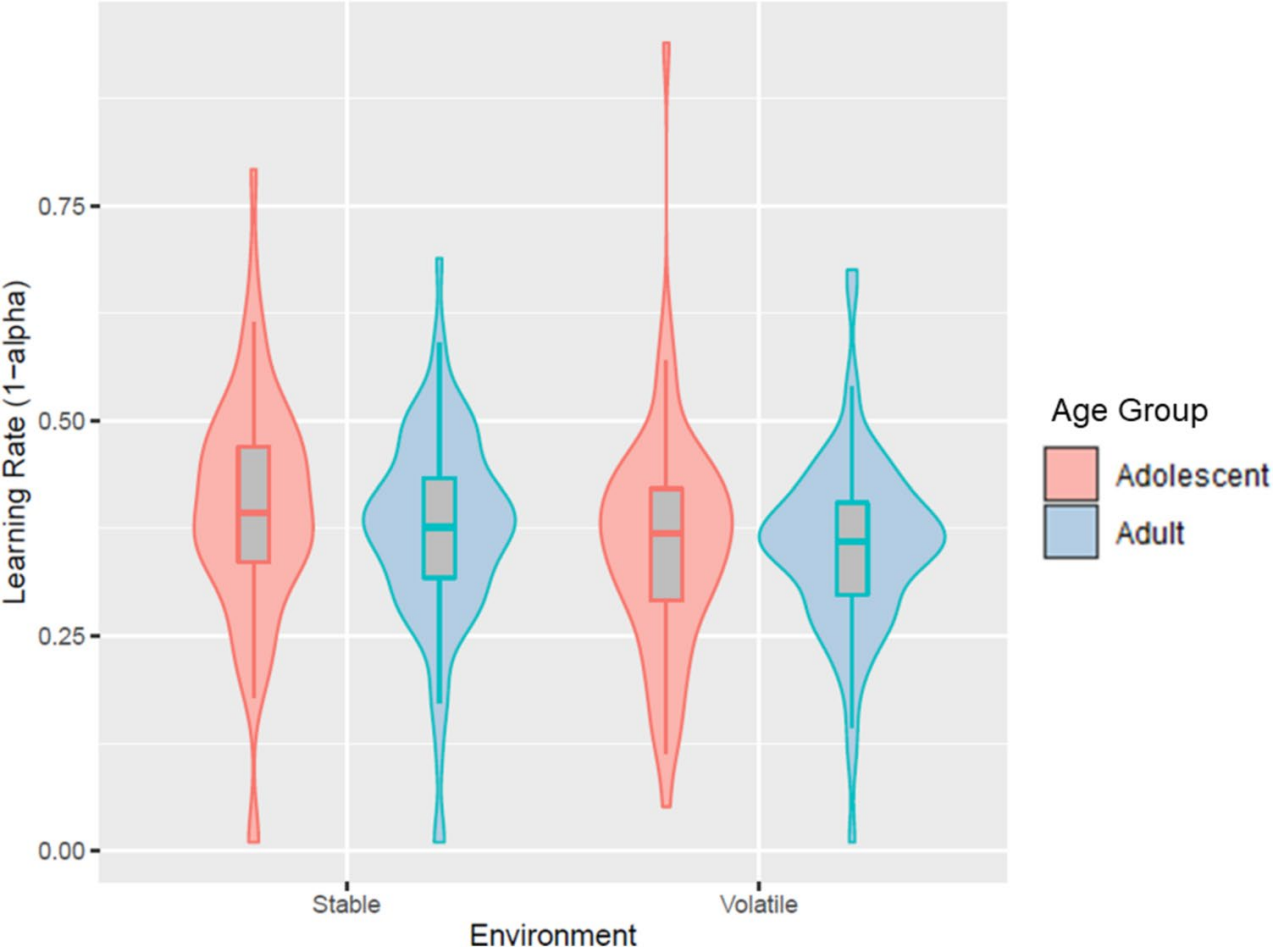
Violin plot demonstrating the absence of evidence for our first preregistered hypothesis. Participants utilised a higher learning rate in the stable environment relative to the volatile environment. The middle bar of each boxplot indicates the median for each age group and the upper and lower bars indicate the upper and lower quartiles, respectively. Whiskers indicate the minimum and maximum values. The violin plot is a kernel density fit to the distribution of data points, with greater width indicating more datapoints are distributed around this value on the y-axis

Because adolescence is a period of heightened cognitive flexibility compared with other age groups (Crone & Dahl, 2012), our first preregistered hypothesis was that relative to adolescents, adults would exhibit a smaller adjustment difference in learning rate between the two environments whereas adolescents would better resemble an optimal RL agent by exhibiting a larger difference (see Supplementary Materials for details confirming that the optimal RL agent increases its learning rate in the volatile compared with stable environment). Yet, we found no evidence for our first pre-registered hypothesis: There was no interaction between environment and age group on learning rate (*F*_(1,138)_ = 1.15, *p* = .286). We did find evidence that participants adjusted their learning rate between the stable and volatile environments (*F*_(1,138)_ = 10.73, *p* = .001, η^2^_*p*_ = 0.07). However, this effect was in the *opposite* direction to our predictions, such that participants utilised a higher learning rate in the stable environment relative to the volatile environment (Fig. 3). We did not detect a difference between adolescents’ and adults’ learning rates (*F*_(1,138)_ = 1.04, *p* = .310).

We also found no evidence for our second pre-registered hypothesis: that anxiety would impair participants’ ability to adjust their learning rate between the volatile and stable environments. An ordinary least squares (OLS) regression demonstrated no evidence that the degree to which participants adjusted their learning rate was predicted by anxiety (β = 0.04, *t* = 0.50, *p* = 0.618). The regression model was not significant (*F*_(1,136)_ = 0.250, *p* = .618, R^2^ = 0.002).

#### Participants adapt to environmental volatility by adjusting stochasticity

In exploratory analyses, we examined whether participants adjusted their stochasticity between the stable and volatile foraging environments. The stochasticity parameter represents the degree to which participants randomly deviate from the option that, to their knowledge, has the highest expected value. Recent work has suggested that stochasticity can be beneficial in environments with changing stimulus-outcome contingencies (i.e., volatile environments), because it can increase exploration of novel options and assist individuals to identify changes to the environment faster than exploiting the option the decision-maker believes to have the highest expected value (Wilson et al., 2021). Our simulations confirmed this account, as the optimal RL agent utilised more stochastic choices in the volatile environment relative to the stable environment (see Supplementary Materials).

**Fig. 4:**
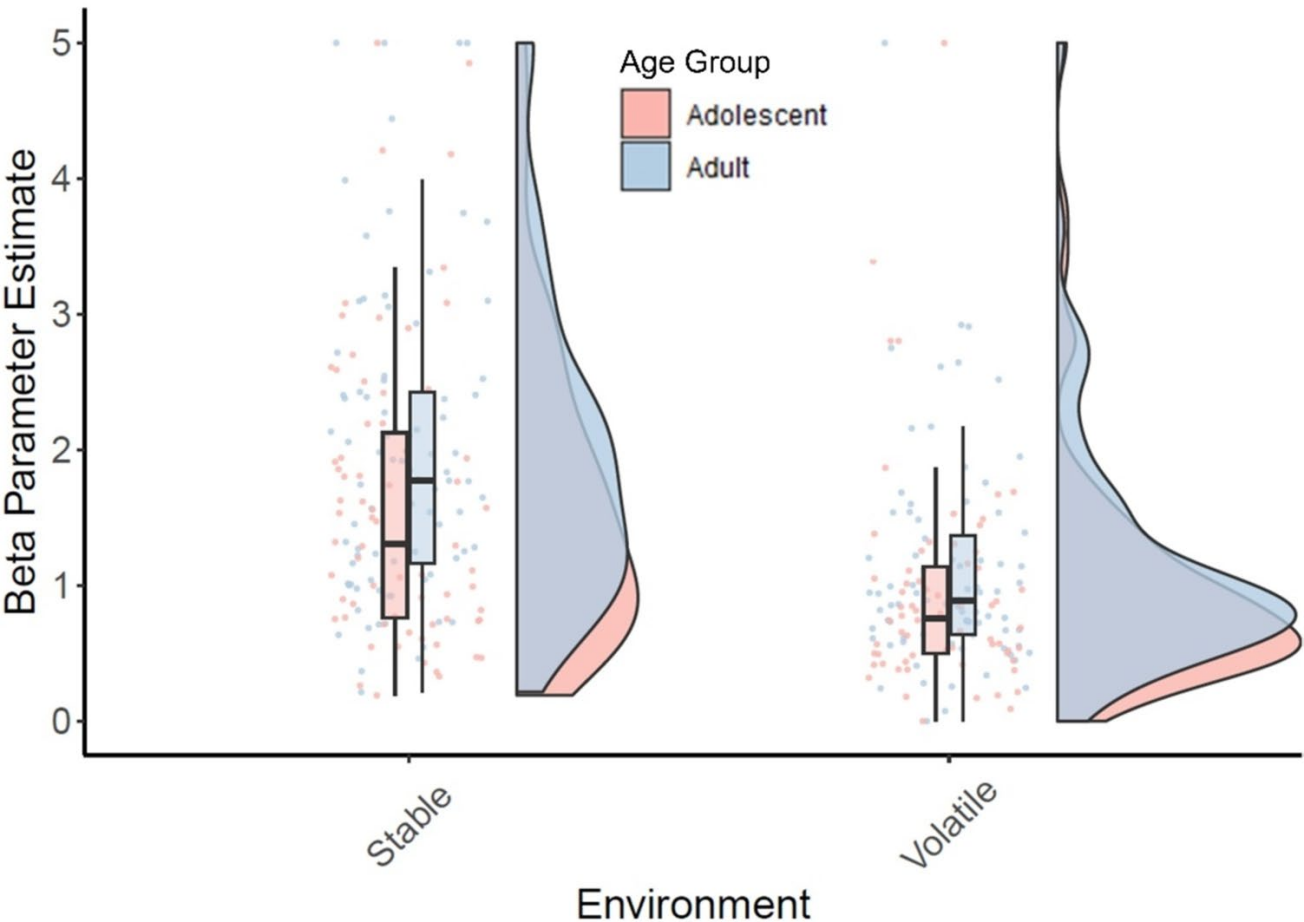
Raincloud plot demonstrating that participants utilised a more stochastic decision-making policy (i.e., lower values on β) in the volatile condition relative to the stable condition and that adolescents were more stochastic than adults in both environments

Consistent with our simulations of an optimal RL agent, participants exhibited more stochastic behaviour in the volatile condition relative to the stable condition (*F*_(1,138)_ = 66.07, *p* < .001, η^2^_*p*_ = 0.32; Fig. 4. Note: Lower β values on this plot indicate more stochastic choices). Simple main effects tests confirmed that adolescents (*F*_(1,138)_ = 21.95, *p* < .001) and adults (*F*_(1,138)_ = 48.89, *p* < .001) each separately exhibited more stochastic behaviour in the volatile environment compared with the stable environment. Furthermore, there was a main effect of age group (*F*_(1,138)_ = 4.35, *p* = .039); adolescents on average exhibit more stochastic decision-making compared with adults. There was not an interaction between foraging environment and age group on stochasticity (*F*_(1,138)_ = 0.65, *p* = .421). We further found that participants who utilised more stochastic choices in the volatile environment relative to the stable environment collected more rewards in the task (*r*(138) = 0.19, *p* = .022). This finding suggests that adjusting stochasticity, rather than learning rate, was an effective computational policy to adapt to the prevailing volatility of foraging environments.

**Fig. 5:**
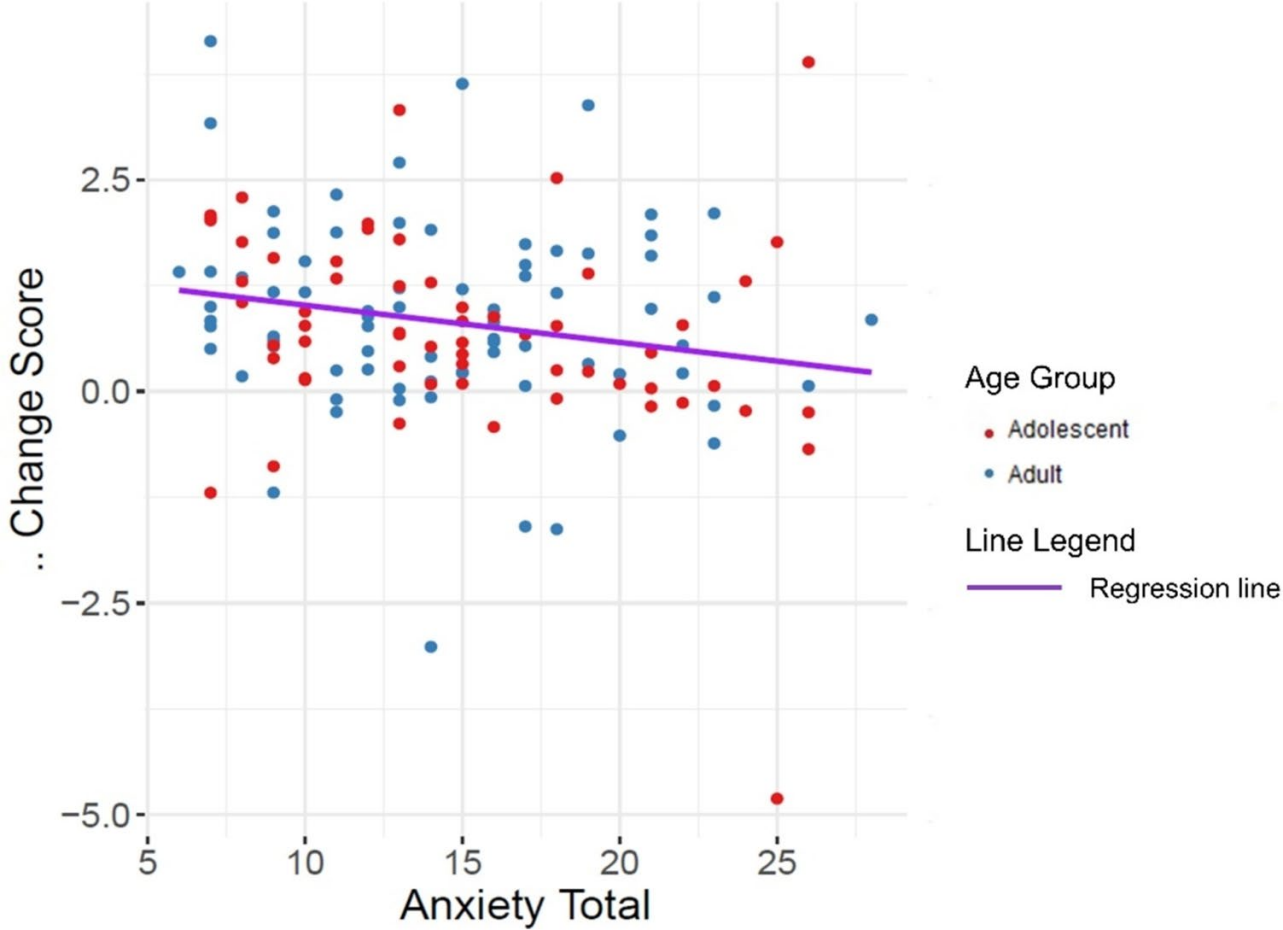
Plot demonstrating the deleterious influence of anxiety (x-axis) on participants’ ability to adjust their decision stochasticity (y-axis) to the prevailing volatility of the environment. β change scores (i.e., the degree to which participants adjust their decision stochasticity) are calculated by subtracting values of β in the stable environment from β in the volatile environment. Values above 0 on the y-axis indicate participants responded more stochastically in the volatile environment relative to the stable environment whereas values below 0 indicate participants who had responded more stochastically in the stable environment. The purple line is the regression line for both age groups, which illustrates how adjustment of stochasticity decreases and thereby departs from optimal adjustment as anxiety increases

We originally hypothesised that anxiety would disrupt participants’ ability to adjust their learning rate to the volatility of the environment (Hypothesis 2). However, we instead found that participants optimise their decisions for environmental volatility through adjusting their stochasticity. We tested whether our original hypothesis about effects of anxiety on the learning rate might hold for stochasticity instead, and our exploratory analyses confirmed that anxiety influenced the degree to which participants adjusted their decision stochasticity between environments. Using the difference in participants’ stochasticity between the volatile and stable environments as an outcome variable and anxiety as a predictor in an ordinary least squares regression, we found that the model was significant (*F*_(1,137)_ = 5.80, *p* = .017, R^2^ = 0.04). This finding suggests that adjusting stochasticity, rather than learning rate, was an effective computational policy to adapt to the prevailing volatility of foraging environments (Fig. 5).

**Fig. 6:**
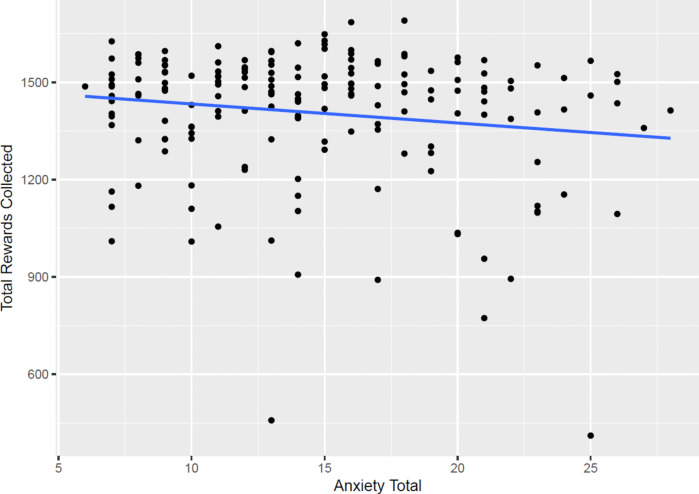
Plot demonstrating the negative association between anxiety and summed rewards collected across both environments. The optimal forager should be able to collect approximately 800 rewards in the stable and volatile environments

To further investigate the deleterious effects of anxiety on foraging performance, we conducted exploratory analysis to examine whether the ability to collect rewards while foraging was impeded by anxiety. Across age groups, there was a negative association between the number of rewards collected in both environments and self-reported anxiety, demonstrating that participants with greater anxiety collected fewer rewards on the task (*r*(174) = −0.15, *p* = .041; Fig. 6). These results were robust to the exclusion of participants with extreme low number of rewards collected (<600 across both environments; see Supplementary Materials).

## Discussion

The present study examined age-related differences in the ability to flexibly adjust foraging behaviour between conditions of stability and volatility. We replicated findings that adolescents explore more than adults in stable foraging environments (Lloyd et al., 2021). We designed our volatile and stable environments such that an optimal RL agent would use a higher learning rate (e.g., more dependence on recent reward feedback) and more stochastic responding (i.e., random deviations from the response with highest expected reward) in the more volatile environment. However, contrary to our hypotheses and previous studies using RL tasks, we found that adolescents and adults exhibited lower learning rates in the volatile environment relative to the stable environment. In contrast, both age groups exhibited more optimal calibration of their decisional stochasticity to the prevailing volatility of the environment, consistent with an optimal RL agent. Such adjustment allowed participants to collect more rewards while foraging, although the ability to adjust stochasticity between stable and volatile environments was impaired by anxiety. Together, these findings identify stochastic responding as a key strategy that optimises human exploration of volatile environments.

Our data did not support our *a priori* prediction that participants would adopt a higher learning rate in the more volatile environment. This prediction derived from research that had attributed adults’ successful decision-making in conditions of volatility to how much weight they give to recent information relative to more historic feedback (i.e., how much they adjust their learning rate; Behrens et al., 2007; Browning et al., 2015). These seminal findings have led to the development of a variety of models utilising dynamic learning rates to account for changing environmental volatility (Piray & Daw, 2021). Yet, we found that participants utilised a *lower* learning rate in volatile environments compared to stable environments, which was inconsistent with previous research and our optimal RL simulation. A lower learning rate for the volatile environment compared to the stable environment may reflect a heuristic to weigh positive reward feedback higher than negative reward feedback while foraging (Garrett & Daw, 2020). This bias could explain our findings, as it would suggest participants have a poorer ability to update their predictions (i.e., use a lower learning rate) in environments that become poorer compared with those that improve in quality (Garrett & Daw, 2020; Xia et al., 2021). While we tried to fit a model with separate learning rates for positive and negative prediction errors, we found this model to be a poorer fit to the data and therefore did not examine this possibility in the present study, though future research could consider this alternative explanation. Another potential explanation of these findings is that the model we implemented was unable to detect changes to participants’ learning rate as some values within the parameter space were less-well recovered in our parameter recovery (see Supplementary Materials). This feature of the model may have precluded our ability to observe changes to participants’ learning rates between the stable and volatile environments. To overcome this limitation, future research could further develop the models used to explain participants’ foraging choices in volatile environments.

Our findings are consistent with accounts that suggest that optimal exploration in conditions of volatility is associated with introducing stochasticity into decision-making (Gershman, 2018). The use of a stochastic decision-making policy, also known as “random exploration,” leads the individual to occasionally divert from the option that, to their knowledge, should have the highest expected value (Wilson et al., 2021). In conditions of volatility, the reward probabilities associated with actions change over the duration of the environment, and such changes affect which action objectively yields the greatest return of rewards. Using a decision-making strategy where the individual occasionally diverts from selecting the option they perceive to have the highest expected value (i.e., exhibiting more stochastic behaviour), the individual can identify changing stimulus-outcome contingencies, faster than if they were to deterministically exploit the option that they perceive to have the highest expected value (Gershman, 2018). Our simulations provide formal support for this account, as we demonstrated that an optimal RL agent utilises greater stochasticity in the volatile foraging environment relative to the stable one. Consistent with this account of optimal exploration, we found that participants rely on a more stochastic decision-making policy in conditions of volatility relative to stability. The model used in the present study assumes that the participants’ decision to stay with their patch is informed by the difference between the reward on their current patch and their estimate of the average rewards available in their environment (the background reward rate), consistent with MVT (Charnov, 1976). This difference informs the probability the participant will stay with their current patch, and our findings suggest that adaptation to volatile foraging environments requires introducing greater stochasticity into participants’ choices. Future work should examine the role of stochasticity in learning the value of patches in environments with multiple patch types (see Harhen & Bornstein, 2023). This recent work has demonstrated that the overexploitation bias observed in adults (Constantino & Daw, 2015) arises from how participants learn the structure of their foraging environment (Harhen & Bornstein, 2023). An interesting direction for future research would be to consider whether stochasticity supports participants to learn the structure of their environment when the reward statistics of individual patches are volatile.

In the present study, we interpret the β parameter to reflect decision stochasticity (as in previous research Chierchia et al., 2023; Nussenbaum & Hartley, 2019). However, an alternative interpretation of the model findings is that the β parameter could instead reflect a poorer ability of the model to capture participants’ behaviour. Specifically, the β parameter could reflect a mismatch between the value computed by the model and the subjective value participants use to estimate when to leave their current patch (Jepma et al., 2020). This interpretation of the β parameter could explain why estimates of this parameter were lower in the volatile environment, as well as lower for adolescent participants relative to adults. However, we note that model fit estimates (AIC and BIC values) did not differ between adolescents and adults, providing some evidence against this interpretation. Furthermore, our simulations demonstrated that participants should use more stochastic choices in the volatile environment independently of participant’s choices.

Importantly, we found that adolescents’ and adults’ ability to increase the amount of stochasticity from stable to volatile environments was impaired by anxiety. It has been demonstrated that anxiety can also impair other features of decision-making, such as individuals’ ability to increase their learning rate from stable to volatile conditions (Browning et al., 2015). One possible reason why we did not find that anxiety affected participants’ ability to adjust their learning rate is because we utilised a foraging task with reward feedback, whereas Browning and colleagues (2015) utilised an instrumental learning task with aversive feedback, specifically an electric shock. Previous research has causally implicated dopamine in controlling the parameter measuring stochasticity in conditions of positive reward feedback but not of aversive feedback (Eisenegger et al., 2014). The association between anxiety and impairments to the dopaminergic reward system (Zweifel et al., 2011) could explain why we observed the effects of anxiety on participants’ ability to adjust their stochasticity. Our findings therefore suggest that anxiety impacts computational decision-making mechanisms (i.e., stochasticity), which are used when tracking the background reward rate. The deleterious effects of anxiety on exploration-based decision making are especially important during developmental periods, such as adolescence, because the individual undergoes significant physical, social, and neurocognitive changes (Eccles et al., 1993; Sawyer et al., 2018; Somerville et al., 2010). However, anxiety is not the only psychopathology associated with biases in explore/exploit decision-making (Lloyd et al., 2024). Future research should consider transdiagnostic symptom profiles that may disrupt the ability to adapt decision-making between stable and volatile environments.

Adolescents’ reliance on more stochastic exploration strategies relative to adults could also account for the increase in novelty-seeking behaviours during this developmental period (Lloyd et al., 2023; Romer et al., 2017). Stochastic or random exploration strategies lead the individual to trial a greater range of behaviours relative to decision-making strategies that rely on rigidly selecting the option perceived to have the highest value (Gopnik et al., 2020). Therefore, adolescents’ increased novelty-seeking behaviours compared to other age groups may be associated with the use of stochastic exploration strategies to negotiate their changeable environment. Indeed, the heightened novelty-seeking associated with stochastic exploration strategies may serve a developmental purpose as a recent study has demonstrated that a decrease in stochasticity from childhood to adulthood allows the organism to trial a range of options in early development and converge on those that yield the greatest reward as the organism reaches maturity (Giron et al., 2023; Lloyd et al., 2023). Adolescents’ novelty-seeking may be driven by the hyperactivity of the dopaminergic reward system during this developmental period (van Duijvenvoorde et al., 2016), which is positively associated with stochastic exploration (but not learning rate; Cinotti et al., 2019; Gershman & Tzovaras, 2018). In contrast to accounts that attribute adolescents’ novelty-seeking to an increase in risk-taking (Steinberg et al., 2008), our findings suggest that the rise in some novel and potentially risky behaviours may be associated with the strategies adolescents use to explore and thereby adapt to their changeable, volatile environment.

Yet, an alternative interpretation of the finding that adolescents exhibit increased exploration is that this behaviour arises from reduced uncertainty aversion in this age group, relative to adults (van den Bos & Hertwig, 2017). In the present study, novel patches had an uncertain reward value, as the initial richness of patches were generated probabilistically. Adolescents’ greater rates of exploration, relative to adults, therefore could reflect reduced aversion to patches with an uncertain reward value, whereas adults preferred to remain with patches with a known but diminishing reward value. Indeed, future research could arbitrate between these two explanations by examining age-related differences in exploration in foraging tasks in which the initial richness is probabilistically generated, which would measure uncertainty aversion, against tasks where the initial richness is fixed, where there is no such uncertainty in the value of new patches (e.g., Experiments 1A and 1B in Constantino & Daw, 2015).

Using a novel task measuring foraging choices in a volatile environment, where stimulus-outcome contingencies are changeable, we have demonstrated that adolescents are better able to adjust their leaving threshold between stable and volatile environments compared with adults. This finding is consistent with the view that adolescents have greater behavioural flexibility compared with adults (Crone & Dahl, 2012), which can support them to navigate their changeable environments. Previous research has demonstrated that adolescents are more adept decision-makers than adults in volatile conditions (Eckstein et al., 2022). Our findings extend this line of research by demonstrating that such an adaptive ability extends to a decision-making context where exploration is determined by estimating the *background reward rate* of the environment (Gabay & Apps, 2021), rather than the value of individual response options measured in n-armed bandit tasks (Christakou et al., 2013; Master et al., 2020; Nussenbaum et al., 2022). The ability to adjust exploration in response to a volatile background rate of rewards may be particularly important for adolescents, because it can allow them to respond to the changeability in opportunities available to them. For example, an adolescent’s decision to stay with their current social group may depend on the availability of other groups available to socialise with. In cases where the quality of available social groups is rapidly changing (e.g., at the beginning of high school or during a summer camp), increases or decreases to the perceived value of these alternative social groups may influence whether the adolescent stays with their current social group. The ability to respond to changes to the background reward rate is an important skill for adolescents to navigate their volatile social and educational environment (Sawyer et al., 2018).

This study also contributes to the growing literature examining the development of foraging strategies across the lifespan (Lloyd et al., 2021, 2022, 2023; Bach et al., 2020). We replicated findings (Lloyd et al., 2021) that show that adolescents explore more than adults and that this heightened exploration is closer to the optimal leaving threshold prescribed by Marginal Value Theorem (Charnov, 1976). The finding that adolescents explore more optimally than adults could indicate that heightened novelty-seeking during adolescence is not primarily associated with negative outcomes (such as dangerous driving or substance misuse; Willoughby et al., 2014), as members of this age group were better than adults at determining the appropriate amount of exploration in the explore/exploit trade-off. As such, the rapid development of the dopaminergic reward system at the onset of puberty (Shulman et al., 2016), which promotes exploratory behaviour (Costa et al., 2014; Gershman & Tzovaras, 2018), may confer benefits in this decision-making context.

One limitation of the present study is that adults’ and adolescents’ data were collected under different conditions. Adolescents completed the task in a classroom environment, whereas adults completed the task at home. While these testing conditions were necessitated by lockdowns introduced in response to the COVID-19 pandemic, we recognise that differences in testing conditions may have affected participants’ foraging behaviour. However, we found no difference in adults’ leaving thresholds in the present study, where data was collected online, and a previous study where data was collected in-person (Lloyd et al., 2021; see Supplementary Materials). A further limitation is that our adolescent sample was limited to 16–17-year-olds, whereas our adult sample included a much wider age range (aged 24–50). While this choice was motivated by theoretical and methodological reasons highlighted above, this group-based design may reduce the sensitivity of the present study to detect environmental volatility effects within the adolescent group, as well as age-effects between the adolescent and adult groups. To overcome this limitation, future research should recruit a wider age range of participants and conduct analyses with age as a continuous predictor of foraging choices across development. Such work would also illuminate whether exploration while foraging exhibits a linear decline from childhood to adulthood or exhibits a quadratic pattern across development.

## Conclusions

The present study has demonstrated that adolescents and adults are able to flexibly adjust their exploration strategies between stable and volatile foraging environments. Specifically, both age groups calibrated the amount of stochasticity in their decision-making to the volatility of the environment, consistent with an optimal RL foraging agent. Importantly, the adaptive ability to adjust stochasticity in decision-making was modulated by anxiety, highlighting features of decision-making that can be negatively affected by anxiety. Consistent with our preregistered predictions, we also replicated findings that adolescents explore more than adults while foraging. However, inconsistent with our predictions, participants deviated from an optimal RL model insofar as they utilised a lower learning rate in the volatile environment compared to the stable environment, which may be attributable to biases to weigh positive feedback higher than negative feedback when foraging. Notably, these findings suggest that there are distinct computational strategies responsible for choosing between options encountered serially, as in our task, compared with options encountered simultaneously, as in previous research, which may be more dependent on learning rate (Browning et al., 2015). Together, our findings contribute to our understanding of how exploration is employed strategically by adolescents and adults to negotiate their surroundings, along with the cognitive strategy associated with foraging in volatile environments.

## Supplementary Information

Below is the link to the electronic supplementary material.

Supplementary file1 (DOCX 4.18 MB)

## Data Availability

Data used for this study is accessible at: https://osf.io/dvqy6/.

## Availability of data and materials

The data for the study are available at https://osf.io/dvqy6/?view_only=2aa41fec60434654a2da28115c46961e. The task can be found at: https://app.gorilla.sc/openmaterials/863981.

## Code availability

The code for the study is available at https://osf.io/dvqy6/?view_only=2aa41fec60434654a2da28115c46961e.
