# Supplementary material for: Stochastic decisions support optimal foraging of volatile environments, and are disrupted by anxiety
Source: Cogn Affect Behav Neurosci. 2025 Jan 9;25(3):868–85. doi: 10.3758/s13415-024-01256-y (PMC12130079; doi:10.3758/s13415-024-01256-y)
Supplement: Supplementary file 1 — Supplementary file1 (DOCX 4.18 MB) [file 13415_2024_1256_MOESM1_ESM.docx]

### Supplementary Material: Stochastic Decisions Support Optimal Foraging of Volatile Environments, and are Disrupted by Anxiety

**Contents:**

1. Parameter Recovery and Model Comparison
2. Simulating Optimal Parameter Values
3. Simulating Optimal Leaving Thresholds in the Volatile Environment
4. Analyses of Rewards Collected in the Task
5. Model Predictive Checks
6. Analyses Controlling for Ethnicity
7. Comparing Lab-Based to Online Data
8. Clinical Cut off Scores for Anxiety Questionnaire
9. Analyses Controlling for Counterbalance Order
10. Instructions to Participants
11. Distribution of Participants’ Ages
12. Model Parameters do not Appear to Vary Within Adults
13. Analyses Excluding Extreme Outliers
14. Sensitivity Analyses of Age on Key Outcome Measures

#### **Parameter Recovery and Model Comparison**

*Parameter Recovery for the Single Learning Rate Model*

We conducted parameter recovery to ensure that the model could provide accurate estimates of each parameter. To evidence this, we used parameters estimated from participants’ data and used these values to simulate the patch foraging task in the stable and volatile environments, producing a total of 308 simulations altogether (157 in the stable environment and 151 in the volatile environment, which is the number of participants who had modelling data). Lower and upper bounds on the model were: α {0,1} and β {0,5} for both parameter recovery and parameter estimation. Minimum and maximum values of α were set on conventional values used for this parameter (Lockwood et al., 2020). Minimum values of β were determined by conventional bounds set on this parameter (Lockwood et al., 2020) and maximum values were set based on simulations which demonstrated poor parameter identifiability above five. The simulated foraging environments had the same settings as the task participants completed, such as the depletion rate, travel time between patches, and number of trials between switches in richness in the volatile environment. Parameter estimation was then conducted on the simulated data, and we examined the correlations between these hardcoded and estimated parameters. We found significant positive correlations between hardcoded and parameter estimates for α (*p* < .001), and *β* (*p* < .001). We considered correlation coefficients of 0.6 and above to indicate acceptable parameter recovery, based on previous studies (Fradkin & Eldar, 2023; see Supplementary Figure 1). While parameter recovery for both free parameters was above this threshold, we note that the parameter recovery for particular regimes of the learning rate were somewhat suboptimal (Supplementary Figure 2). Parameter estimates for α and *β* were weakly correlated.


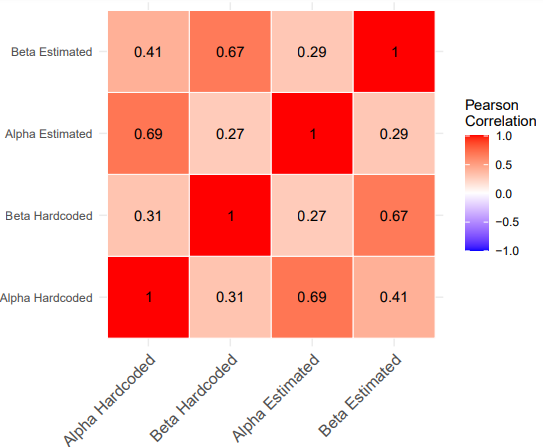


*Supplementary Figure 1: Heatmap demonstrating the hardcoded and estimated parameter values from our computational model. Values in each tile denote the correlation coefficient between variables.*


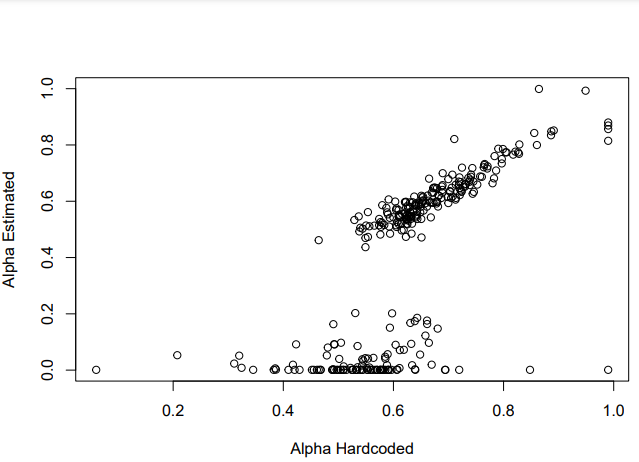

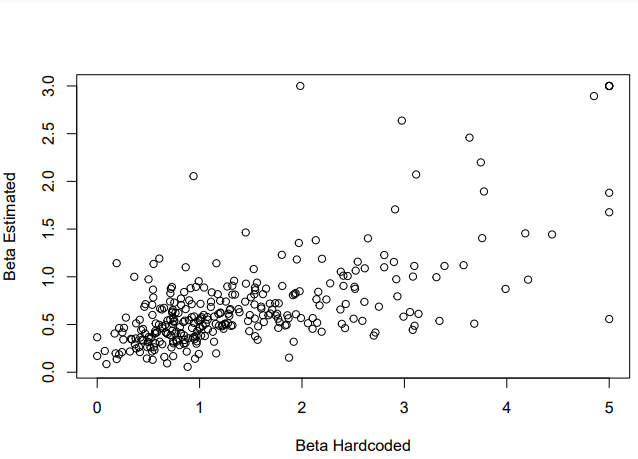


*Supplementary Figure 2: Scatterplots indicating hardcoded parameters (x-axis) and estimated parameters (y-axis) for each of the free parameters.*

*Model Comparison Between Single and Dual Learning Rate Models*

We also compared the formal model described in the main text to a model which separately estimated from participant data learning rates for positive and negative prediction errors (Garrett & Daw, 2020). In this model, we calculated whether the reward feedback experienced by participants is better than expected (i.e., is a positive prediction error), or poorer than expected (i.e., a negative prediction error) using the following formula:

*δ* *_i_* ← *s_i_* /*τ_i_* – *ρ_i_*

When delta (δ) is greater than 0, participants update their estimate of the average reward rate according to Equation (1) described in the main manuscript and the weight placed on this feedback is controlled by the free parameter α_pos_. When delta is less than 0, this feedback is integrated into the participants’ estimate of the average reward rate, controlled by the free parameter α_neg._ The participant’s estimate of the average reward rate is then entered into the SoftMax function described in Equation (2) in the main manuscript. Finally, we also tested a simpler Reinforcement Learning model which included a single learning rate and regular Softmax equation (as opposed to the Softmax equation that incorporated the MVT quantities that was used in the main text).

We conducted model comparison for each of these models, calculating the AIC and BIC of each model (Wilson & Collins, 2019). Lower values on these criteria indicate the model is a better fit to the data (Daw, 2011). Results of model comparison of the AIC and BIC in each foraging environment demonstrated overwhelming support for the model that included a single learning rate for all prediction errors, but not the simpler Reinforcement Learning Model (see Supplementary Table 1). As such, we proceeded with our analyses using this winning model.

*Supplementary Table 1: Average AIC and BIC values for each model for the adolescent and adult groups*

|  | Adolescents | Adults |
| --- | --- | --- |
| Simple RL Model AIC | 161.87 | 169.80 |
| Simple RL model BIC | 167.34 | 175.35 |
| Single Learning Rate Model AIC | 67.66 | 62.64 |
| Single Learning Rate Model BIC | 73.15 | 68.19 |
| Dual Learning Rate Model AIC | 89.14 | 84.16 |
| Dual Learning Rate Model BIC | 97.34 | 92.49 |

#### **Simulating Optimal Parameter Values**

As we were interested in how participants’ behaviour compares to an optimal reinforcement learning (RL) agent, we simulated task behaviour systematically varying the combination of parameter values to identify which parameters yielded the greatest number of rewards. We simulated an RL-informed forager’s behaviour in stable and volatile foraging environments, which reproduced the design of the empirical study. We conducted a grid search across different combinations of free parameters in these environments within the ranges: α {0,1} in increments of 0.1 and β {0,5} in increments of 0.5. These ranges were selected as they resembled the range of values used in the parameter estimation and recovery (see Section 1. Parameter Recovery and Model Comparison). The simulated environments had the same reward statistics as those utilised in the empirical study (see Methods), such as those used for the initial richness of patches, depletion rates and volatility manipulation.

We visualise the resulting simulations as a heatmap, indicating which combinations of parameters yielded the highest rewards. As the heatmaps indicated there was a range of parameter values that produced approximately the same maximal reward values, we plotted combinations that yielded high rewards (those above 780 in the stable environment and those above 850 in the volatile environment) along with histograms that identify the combination of parameters that reliably yielded high rewards. We interpret the parameter values that reliably produce high reward yields as the optimal parameter values in each environment. These simulations demonstrate it is optimal to use a higher learning rate (0.22) in the volatile environment and use a lower learning rate (0.185) in the stable environment. Further, they demonstrate that it is optimal to exhibit more stochastic choices (i.e., lower values of β; 0.23) and use less stochastic choices (i.e., higher values of β; 0.75) in the stable environment (see Supplementary Figure 3).


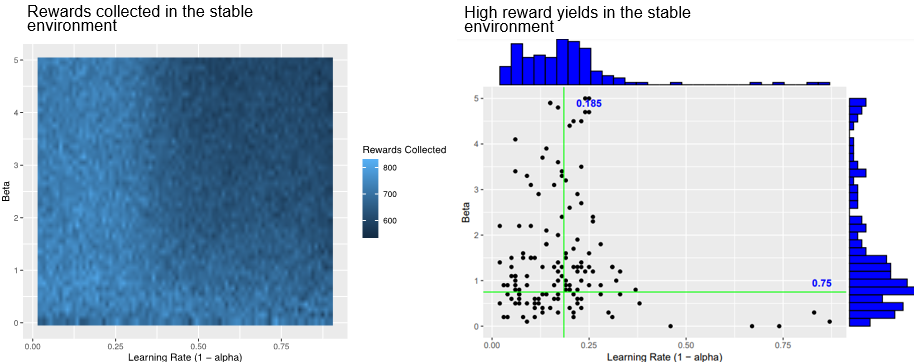
 *
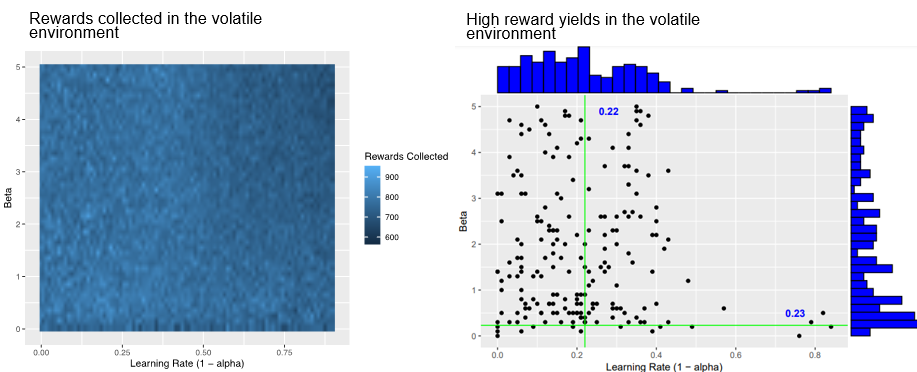
Supplementary Figure 3: Heatmap plots demonstrating the combination of free parameters that yield the highest number of rewards in the stable environment (top two panels) and volatile environment (bottom two panels). Figures on the left indicate the full range of rewards that can be collected using combinations of free parameters. Figures on the right are indicate the density of high-value yields (i.e., 780 or more units of reward in the stable environment and 850 or more units of reward in the volatile environment), with each point representing a combination of parameters that produced high-value yields within each environment. These plots therefore highlight parameter combinations that yield the highest rewards, with precise parameter values annotated within the plot.*

1. **Simulating Optimal Leaving Thresholds in the Volatile Environment**

To simulate the optimal leaving thresholds in the volatile environment, we simulated an agent that used separate leaving thresholds in the rich and poor portions of the volatile environment (ranging from 1-11 in increments of 0.1 in both rich and poor portions). This simulation produced a total of 10,201 simulated agents (see Supplementary Figure 4). We then identified the trial number that yielded the highest number of rewards and inspected the combination of leaving thresholds used in this trial. This process allowed us to identify that the optimal leaving threshold in the rich portion of the volatile environment was 8.9 and the optimal leaving threshold for the poor portion of the volatile environment was 3.65. Note, simulation of the optimal leaving threshold in the stable environment is reported in Lloyd et al. (2021).


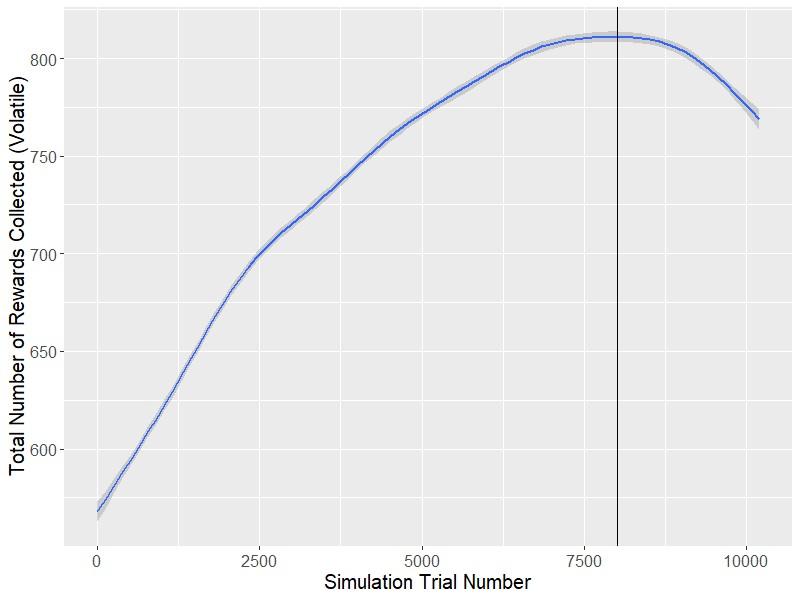


*Supplementary Figure 4: Plot indicating the trial yielding the highest reward value in the volatile environment, determined by combining combinations of leaving thresholds for the rich and poor portions of this environment.*

1. **Analyses of Rewards Collected in the Task**

Although we found that adolescents’ performance was closer to the optimal leaving threshold in the stable environment and that this age group exhibited greater adjustment of their leaving threshold in the volatile environment, we found no evidence that this translated to a greater number of rewards collected on the task. There was not a main effect of age group (*F*_(1,173)_ = .57, *p* = .450) or interaction between age and environment (*F*_(1,173)_ = 0.02, *p* = .890), suggesting no evidence that adolescents and adults differed in the number of rewards they collected across both environments. While this finding may appear inconsistent with the evidence that adolescents’ leaving threshold was closer to the behavioural optimum compared to adults’, we note that the number of points collected is a coarser measure of performance, as rewards were generated probabilistically. As the reward schedule was probabilistic, adolescents may not have received more rewards despite utilising a closer-to-optimal leaving threshold compared to adults. Indeed, previous work which used a greater number of foraging environments did not demonstrate evidence that adolescents collected more rewards despite their leaving thresholds being more optimal than adults’ across every environment (Lloyd et al., 2021). Finally, we found a main effect of environment, as participants collected a higher number of rewards in the volatile foraging environment compared to the stable environment (*F*_(1,173)_ = 12.51, *p* < .001, η^2^ = 0.02; see Supplementary Figure 5).


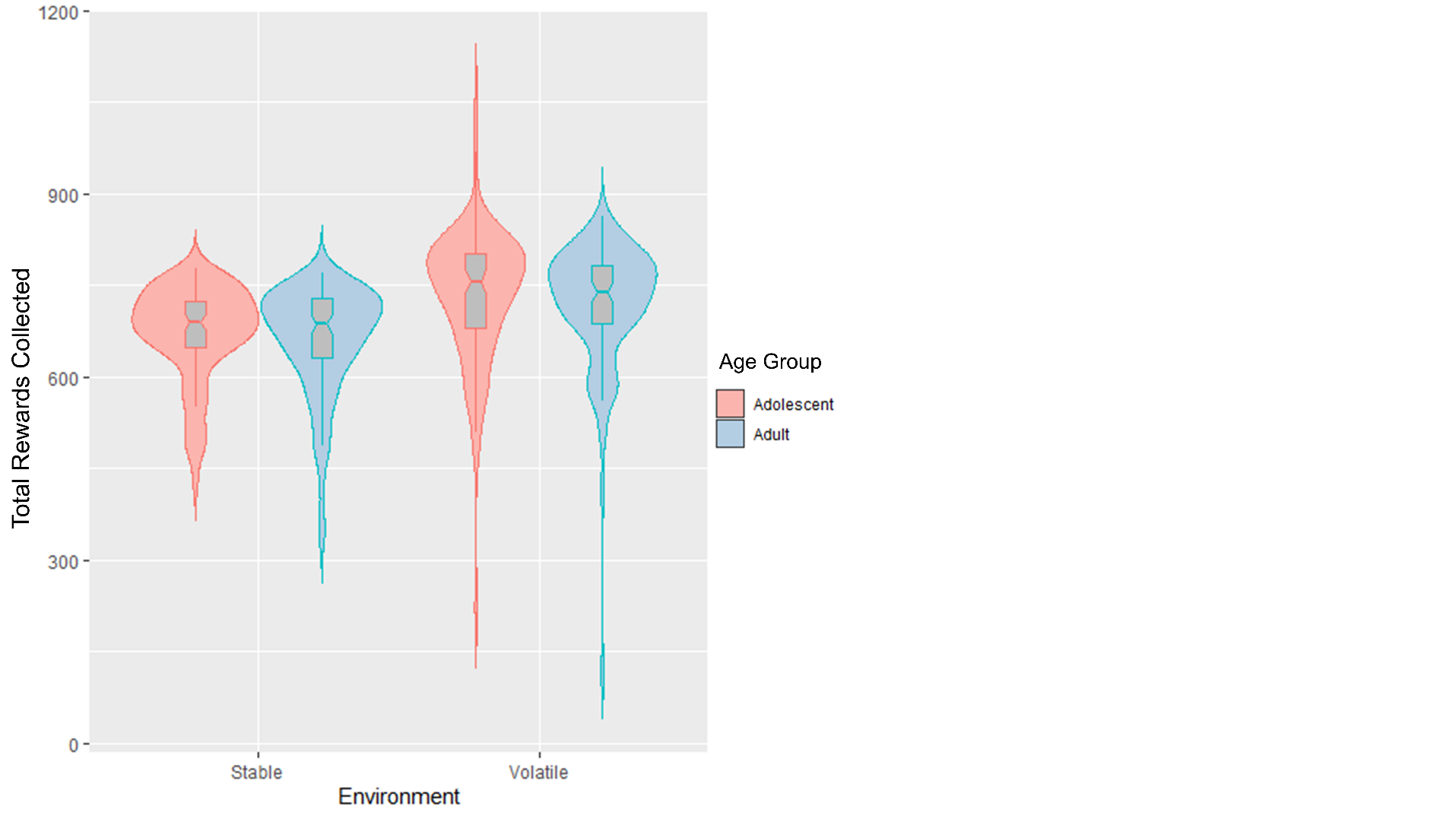


*Supplementary Figure 5: Violin plot demonstrating that adolescents and adults collected a similar number of rewards in both environments, though participants collected more rewards in the volatile environment relative to the stable environment. The boxplots demonstrate the upper and lower quartile. According to our simulation, the optimal forager should be able to collect at least 800 rewards in each environment.*

1. **Model Predictive Checks**

To assess whether our model could recapitulate participants’ explore/exploit choices, we simulated the stable and volatile environments using parameter values estimated from participants’ data. These simulations included versions of the volatile environment where the rich portion was encountered first, as well as when the poor portion was encountered first. These figures demonstrate that the model was able to recapitulate participants’ behaviour in the task, including age-related differences observed such as adolescents’ heightened exploration, relative to adults. However, we did note a tendency for the model to underestimate participants’ leaving threshold in the first ~20 trials of each simulated environment (see Supplementary Figure 6).


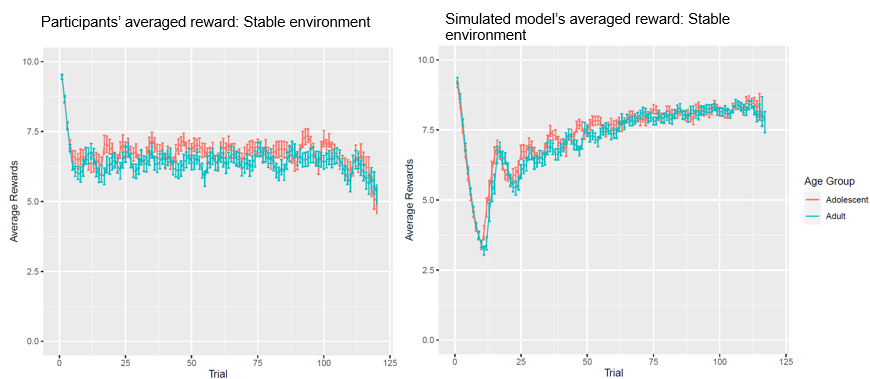


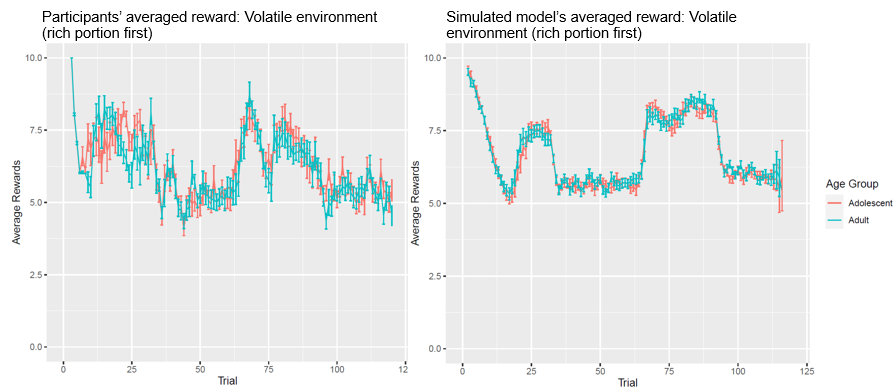


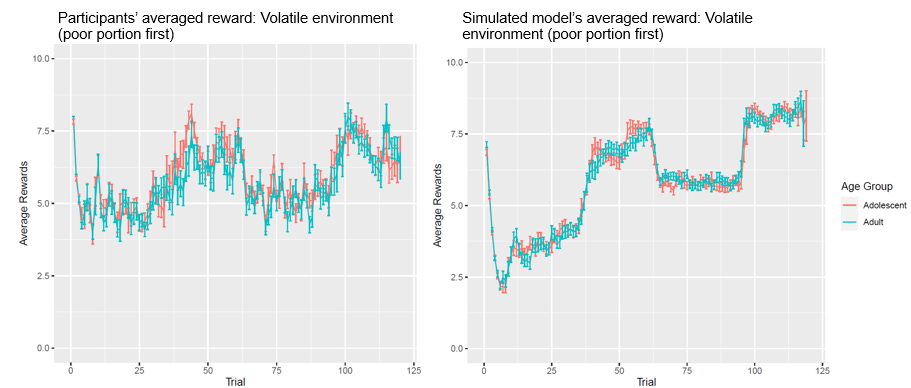
*Supplementary Figure 6: Plots demonstrating simulated data using participants’ parameter values can recapitulate participants’ explore/exploit choices (with error bars). Left plots denote participants’ averaged explore/exploit choices in each environment. The right plots are simulated data using parameter values estimated from participants’ data (right plots), averaged across the total number of simulations (with error bars). The top two plots are the stable environment, the middle two plots are the volatile foraging environment when the rich portion was encountered first, and the bottom two plots and the volatile foraging environment when the poor portion was encountered first. Peaks in the line indicate when participants (or simulated data) encountered new patches, whereas troughs in the line indicate participants’ (or the simulated data’s) leaving threshold.*

**6. Analyses Controlling for Ethnicity**

The adult sample in our study predominantly comprised White (UK) participants, whereas our adolescent sample was more ethnically diverse (see Supplementary Table 2). We therefore conducted additional analyses to ensure our primary results were not confounded by the difference in ethnic diversity between the two groups. Entering participants’ ethnicity as a co-variate in our analyses, we found that our main results were preserved when accounting for this variable.

*Supplementary Table 2. Descriptive statistics of demographic variables for the adolescent and adult samples. Adults reported their own highest level of education whereas adolescents reported their parents’ highest level of education.*

|  | **Adolescent** | **Adult** |
| --- | --- | --- |
| *Participant ethnicity* |  |  |
| White (UK) | 69.32% | 96.59% |
| White (other) | 6.82% | 0% |
| Mixed race | 11.36% | 1.14% |
| Indian | 2.27% | 0% |
| Pakistani | 3.41% | 3.41% |
| Bangladeshi | 1.14% | 0% |
| Chinese | 0% | 0% |
| Asian (other) | 6.82% | 3.41% |
| African | 0% | 0% |
| Caribbean | 1.14% | 1.14% |
| Black (other) | 1.14% | 1.14% |
| Arab | 1.14% | 0% |
| Education | 2.66 | 3.24 |

1. **Comparing Lab-Based to Online Data**

In the present study, adults’ data were collected online whereas adolescents’ data were collected in a classroom. It is possible that these testing environments may have biased adults’ foraging behaviour relative to adolescents. To partially exclude this possibility, we examined whether the data collected online in our adult sample differed systematically to adults’ data collected in lab-based conditions in a previous study (Lloyd et al., 2021). Specifically, the stable environment used in the current study was the same as ‘Environment 1’ in Lloyd et al. (2021). Comparing these datasets, we found no evidence that adults’ leaving thresholds in the data collected online differed to the leaving thresholds of adults data collected under lab-based conditions (*t*(155) = 0.29, *p* = .773). We note that computational analyses were not performed on the data reported in Lloyd et al. (2021) and therefore we cannot compare these datasets on any computational variables.

1. **Clinical Cut off Scores for Anxiety Questionnaire**

We plotted the distribution of scores on the GAD-7 separately for adolescent and adults to examine the distribution of anxiety scores in our samples (see Supplementary Figure 7). We did not find a significant difference between adolescents and adults in their anxiety scores (*t*(174) = -0.10, *p* = .922).


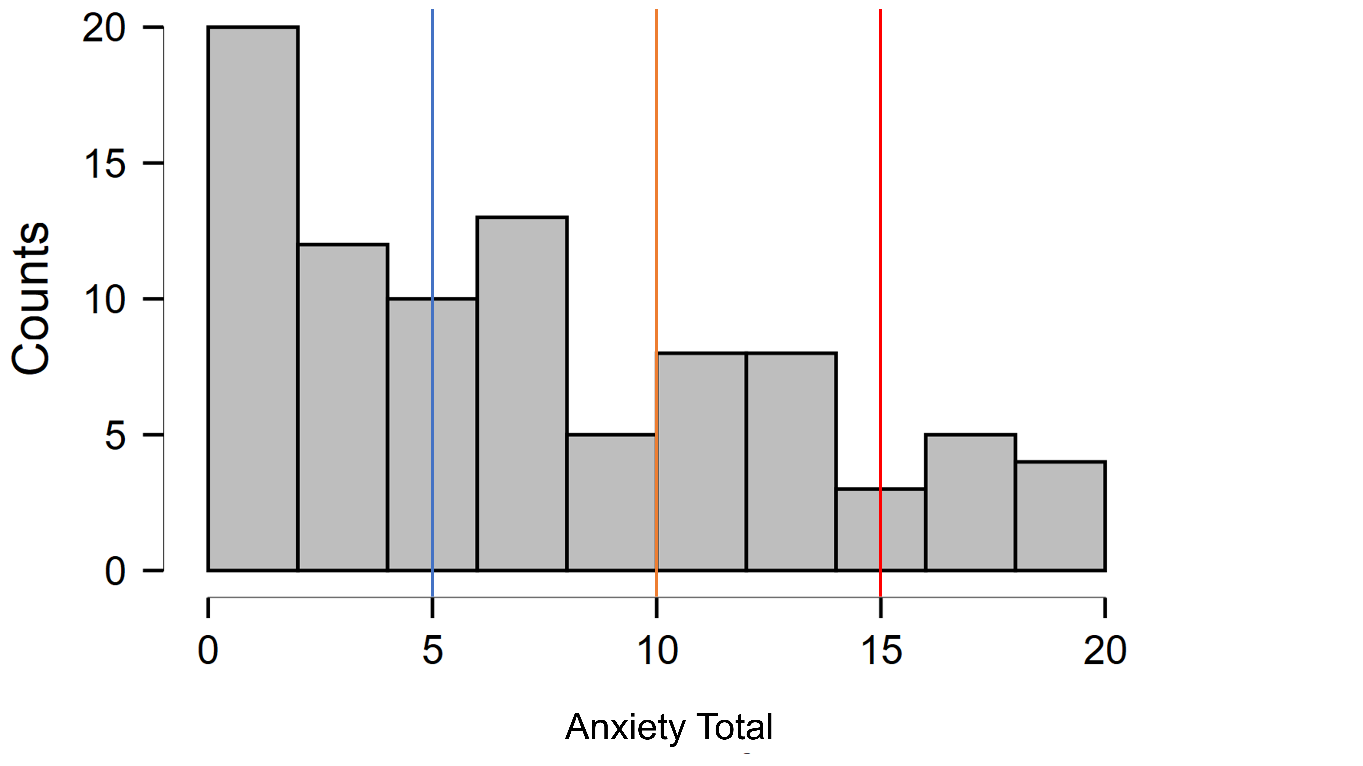


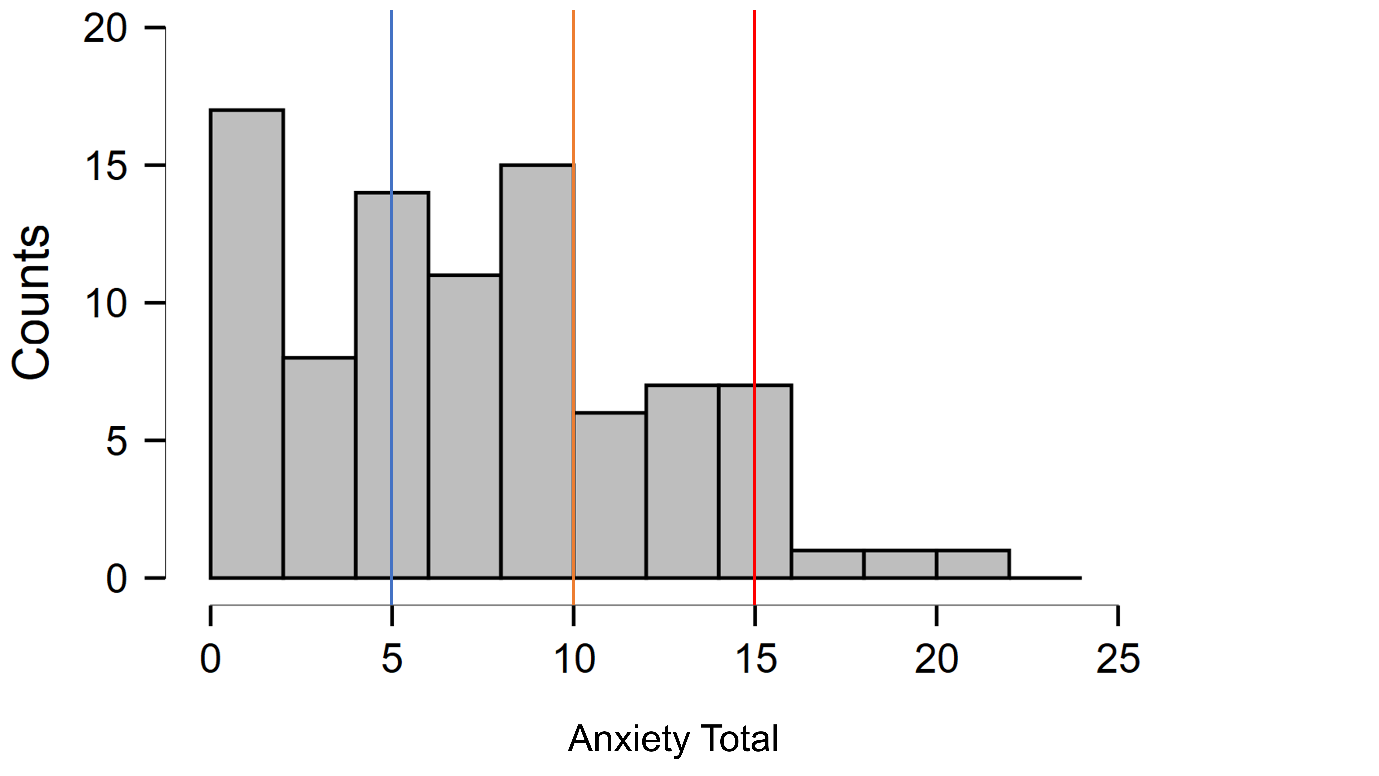


*Supplementary Figure 7: Plots demonstrating the distribution of anxiety scores in adolescents (top plot) and adults (bottom plot). The blue line indicates the clinical cut off for mild anxiety, the orange line indicates the clinical cut off for moderate anxiety, and the red line indicates the clinical cut off for severe anxiety.*

1. **Analyses Controlling for Counterbalance Order**

We conducted analyses controlling for whether participants encountered the stable or volatile foraging environment first to examine whether this influenced our results. To examine effects on leaving threshold, our behavioural measure of participants’ foraging choices, we entered environmental richness (volatile rich, volatile poor, and stable) and age group (adolescent or adult) as factors into a 2*3 repeated measures ANCOVA, with the order in which participants encountered the stable and volatile environments as a covariate. The results replicated those reported in the main text, as we found a main effect of environmental richness on participants’ leaving thresholds (Greenhouse-Geisser corrected; *F*_(1.76,330.02)_ = 28.49, *p* < .001, η^2^*_p_* = 0.14). Adolescents utilised a higher overall leaving threshold demonstrated through a main effect of age group (*F*_(1,172)_ = 7.57, *p* = .007, η^2^*_p_* = 0.04), consistent with previous work (Lloyd et al., 2021). There was an interaction between age group and environmental richness on the degree to which participants adjusted their leaving thresholds (Greenhouse-Geisser corrected; *F*_(1.76,330.02)_ = 4.45, *p* = .016, η^2^*_p_* = 0.03). In addition, there was an interaction (*F*_(1.76,330.02)_ = 3.63, *p* = .033, η^2^*_p_* = 0.02) between environment and the order with which participants encountered the stable and volatile environments, which is consistent with previous work (Garrett & Daw, 2020). Together, these findings indicated that participants’ leaving thresholds did differ based on whether they encountered the stable or volatile environment first, though this did not affect our primary findings that adolescents explored more than adults, nor that participants adjusted their leaving thresholds between the stable environment, rich portion of the volatile environment and poor portion of the volatile environment.

Similarly, when controlling for the order in which participants encountered the environments for the learning rate analyses, the findings replicated those in the main text. Specifically, there was no interaction between environment and age group on learning rate (F_(1,136)_ = 1.53, p = .218), though we did find a main effect of environment type on learning rate (F_(1,136)_ = 14.96, p < .001). There was no a main effect of age group (F_(1,136)_ = 1.28, p = .260), nor counter balance order (F_(1,136)_ = 1.863, p = .175) on participants’ learning rate. However, there was an interaction between environment and counter balance order (F_(1,136)_ = 4.60, p = .034).

Finally, when controlling for the order in which participants encountered the environments for the stochasticity parameter, we replicated the finding that participants adjusted their stochasticity between stable and volatile foraging environments (F_(1,136)_ = 26.14, p < .001), as well as the finding that adolescents utilised more stochastic choices than adults (F_(1,136)_ = 4.06, p = .046). There was not a main effect of counterbalance order on the degree to which participants utilised stochastic choices (F_(1,136)_ = 0.49, p = .484), nor an interaction between environment and age group (F_(1,136)_ = 0.73, p = .394), or environment and counterbalance order (F_(1,136)_ = 0.40, p = .531). Together, these analyses replicate our findings that participants failed to adjust their learning rate to the prevailing environmental volatility and instead adjusted their stochasticity to this change in the environment, which was independent of the order with which they encountered these conditions.

1. **Instructions to Participants**


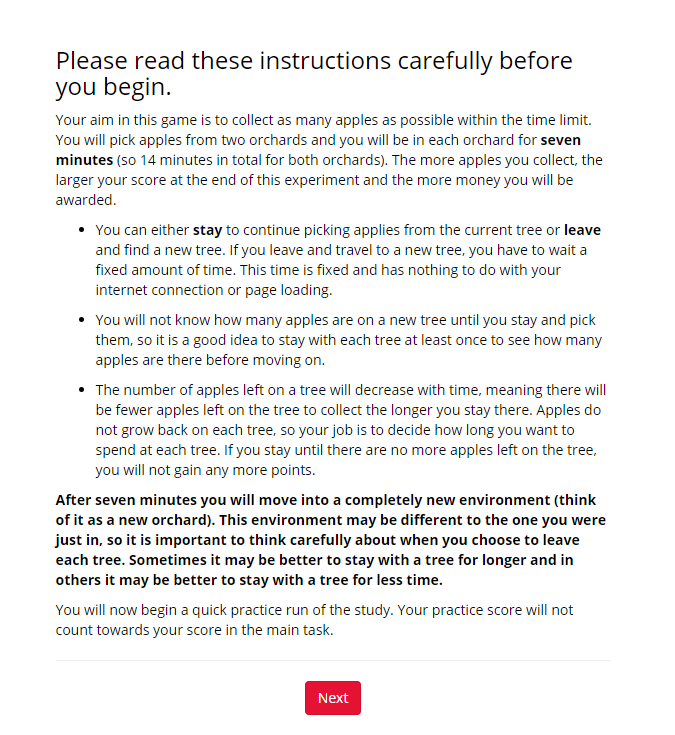


After the practice environment, participants were provided with the following information:


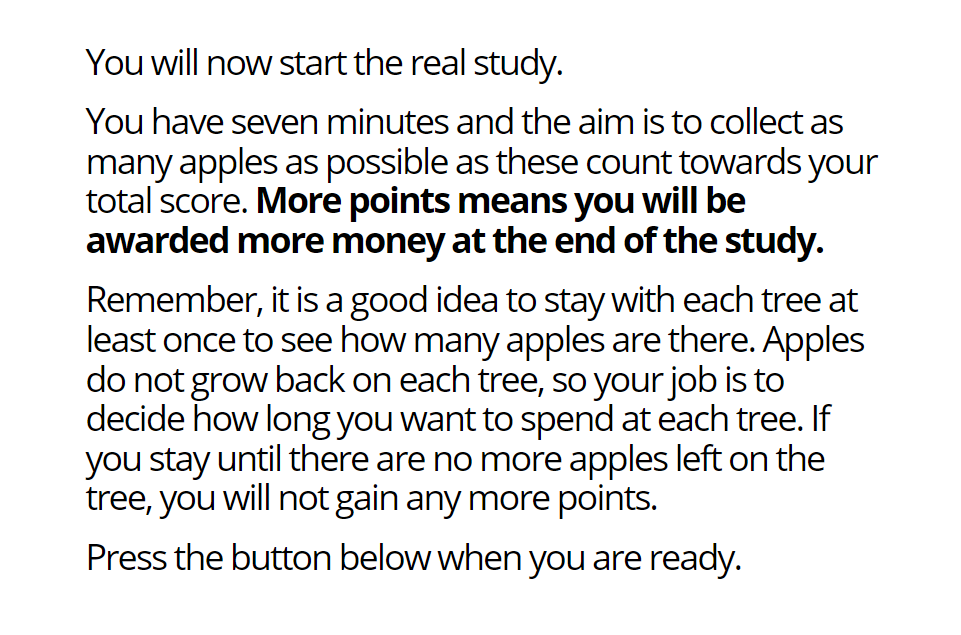


1. **Distribution of Participants’ Ages**


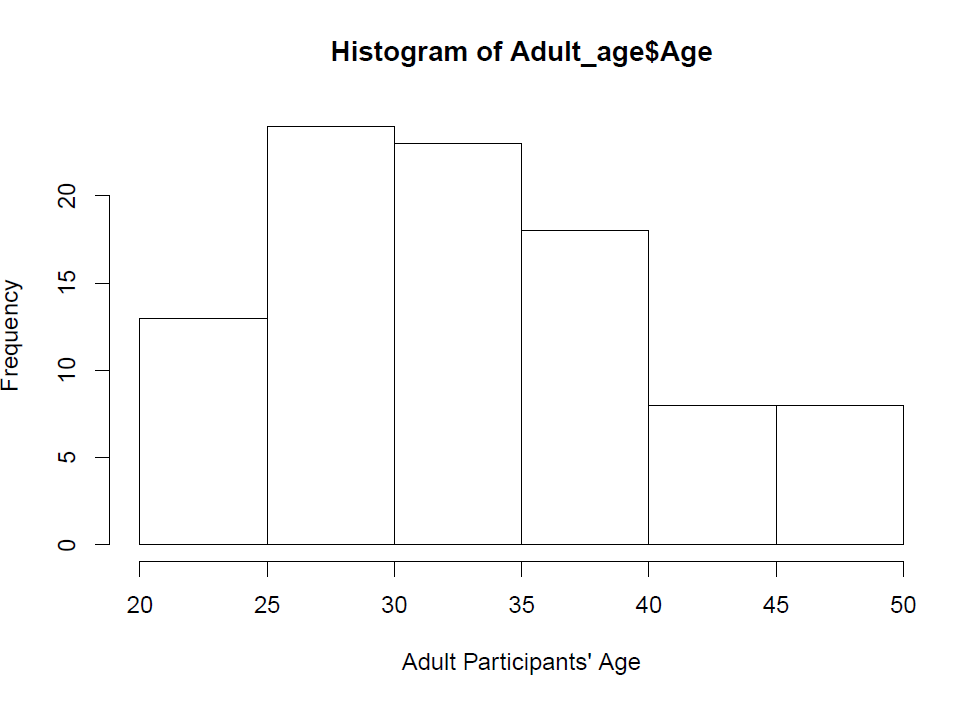


*Supplementary Figure 8: Histogram depicting the distribution of adult participants’ ages, demonstrating the majority of adult participants were aged below 40.*


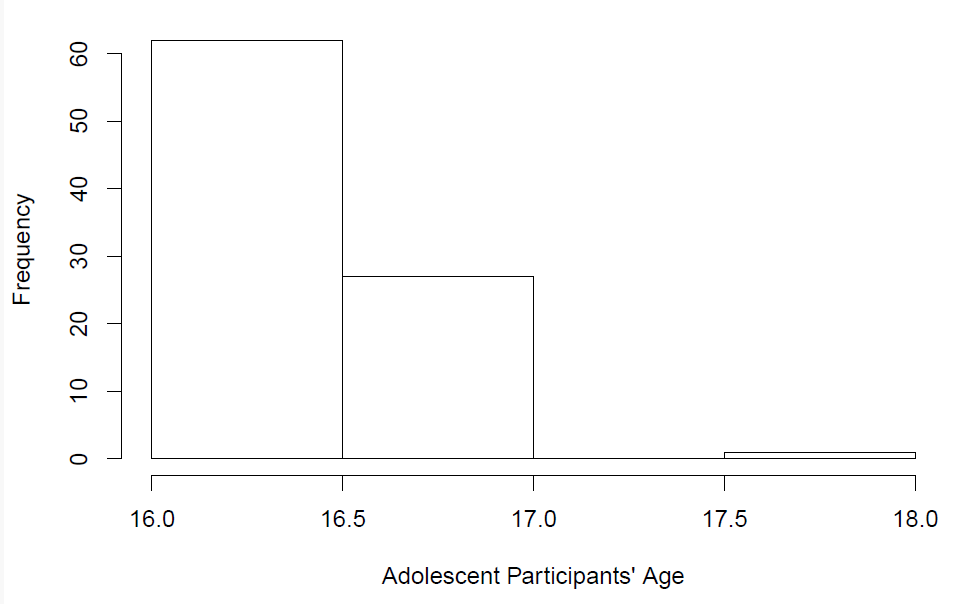


*Supplementary Figure 9: Histogram depicting the distribution of adolescent participants’ ages.*

1. **Model Parameters do not Appear to Vary Within Adults**

Given our decision to treat age as a categorical variable, it was important to exclude the possibility that model parameters varied within the adult sample. Indeed, had this been the case it would have motivated analyses in which age was a continuous predictor of model parameters. To this end, we conducted correlation analyses within the adult sample examining the association between participants’ age and model parameters (α and β) in both stable and volatile environments. These analyses demonstrated that the model parameters were not associated with participants’ age, with the exception of β in the stable environment where we observed a weak positive correlation suggesting that stochasticity declines with age (see Supplementary Figure 10. These findings motivated our decision to collapse adult participants into a single group for the analyses reported in the main text.


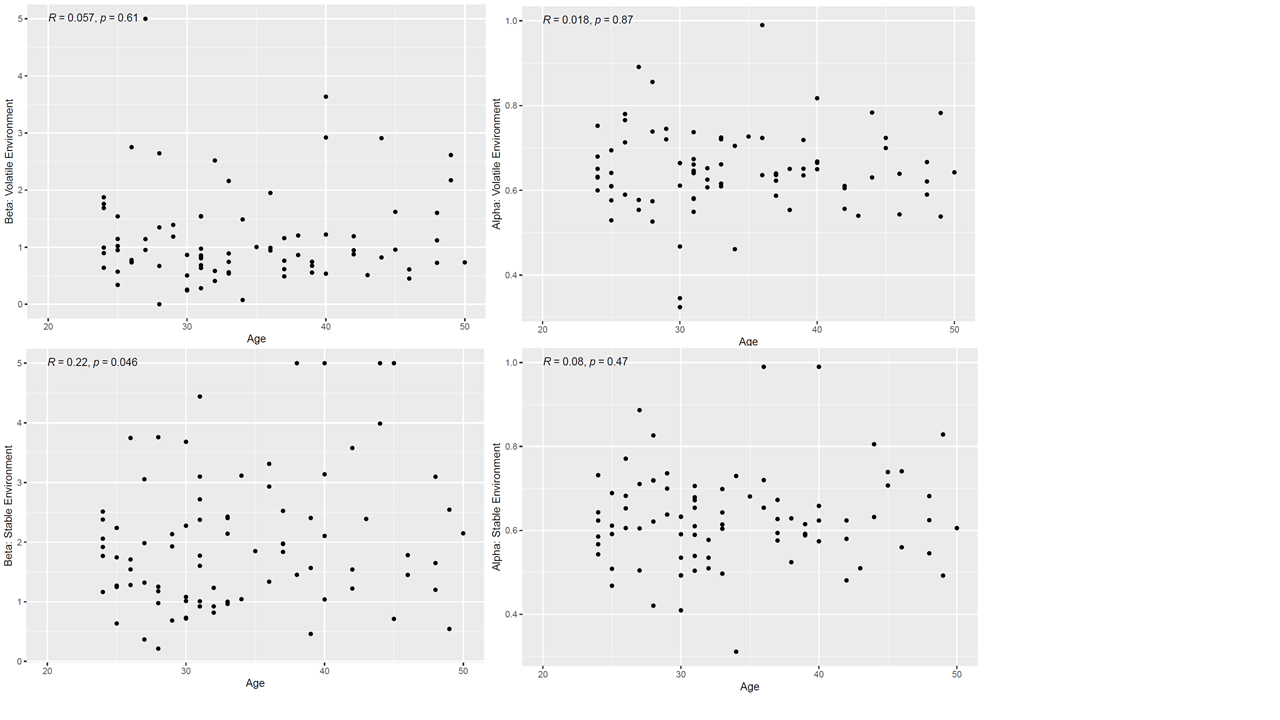


*Supplementary Figure 10: Scatterplots demonstrating the absence of age-related changes to model parameters within the adult age group, with the exception of values of β in the stable foraging environment. The two left hand plots demonstrate the association (or absence thereof) between age and values of β in the stable (bottom left panel) and volatile (top left panel) environments. Plots on the right demonstrate the absence of an association between age and values of alpha in the stable (bottom right panel) and volatile (top right panel). Correlation coefficients (R) and p values for the correlation analyses are displayed in the top left corner of each plot.*

1. **Analyses Excluding Extreme Outliers**

We observed some cases of extreme scores reported for anxiety, as well as on task-based measures including rewards collected in the task and change scores on the β parameter. We examined whether our results were robust to the exclusion of these outliers, retaining a significant association between self-reported and anxiety and rewards collected (r(172) = -0.13, *p* = .047). However, when excluding the participant with an extreme β change score, we no longer found anxiety was a significant predictor of β change scores, nor was the overall model significant (*F*_(1,136)_ = 3.07, *p* = .082, R^2^ = 0.04).

1. **Sensitivity Analyses of Age on Key Outcome Measures**

To exclude the possibility that our findings were driven by specific ages within our adult sample, we conducted sensitivity analyses to examine whether the association between participants’ age and key outcome measures were particularly influenced by certain datapoints (i.e., ages). We first conducted regression analyses entering participants’ age as a continuous variable and patch foraging environment as predictors of key outcomes reported in the main manuscript (specifically leaving thresholds, learning rates and stochasticity). We then calculated the Cook’s D statistic for each regression model, which estimated whether particular datapoints are highly influential in driving the associations found in the regression model. These analyses allowed us to examine whether certain ages drove the difference between adolescents and adults, as reported in the main manuscript. We note that analyses using age as a continuous variable (rather than categorical, as reported in the main manuscript) reproduced our key findings. Further, none of the Cook’s D statistics were above 0.5, which would indicate a datapoint (i.e., that of a specific age) is disproportionately driving the associations in the regression model. We report the findings of these models and the sensitivity analyses below.

Reproducing the findings for participants’ leaving thresholds, we found that age negatively predicted leaving thresholds, such that older participants utilised lower thresholds (β = -0.04, t = 4.35, *p* < .001). Participants’ leaving thresholds were also predicted by the environment (whether this was the stable environment, the rich portion of the volatile environment or the poor portion of the volatile environment; β = -0.32, t = 3.10, *p* = .002). None of the Cook’s D statistics exhibited values above 0.02 (see Supplementary Figure 11).


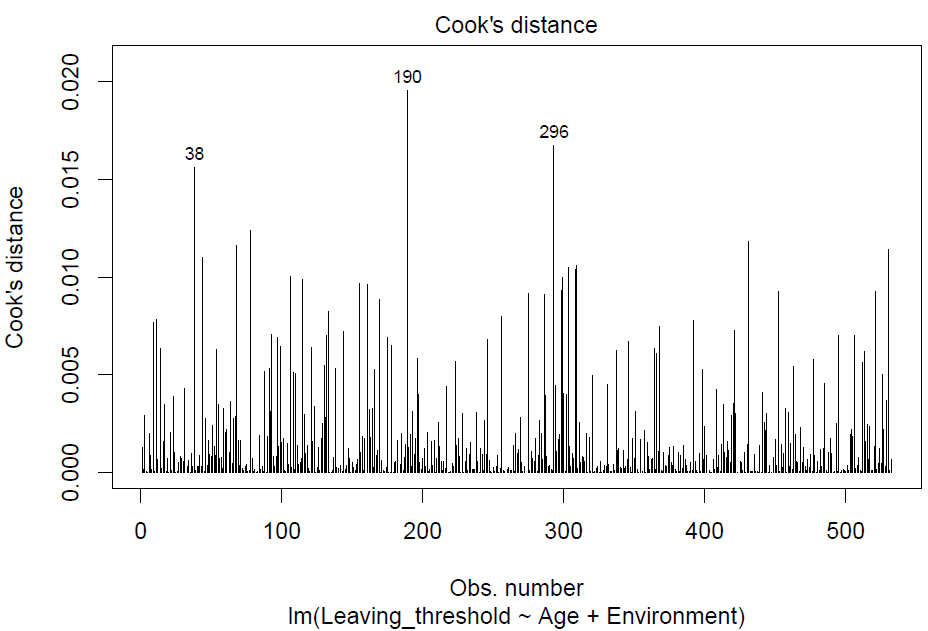


*Supplementary Figure 11: Plots demonstrating the absence of evidence for specific ages disproportionately affecting the association between age and participants’ leaving threshold. The x-axis on these plots indicate the observations, which are higher than the number of participants as there is one observation per participant per environment. The y-axis denotes the Cook’s distance for each datapoint, with values above 0.5 indicating an influential datapoint.*

In addition, we reproduced the finding that age positively predicted participants’ estimates of β, where higher values indicate less stochasticity (β = 0.02, t = 3.05, *p* = .003). Stochasticity was also predicted by environment, suggesting participants utilised more stochastic choices in the volatile environment relative to the stable environment (β = -0.75, t = 6.69, *p* < .001). None of the Cook’s D statistics exceeded values of 0.05 (see Supplementary Figure 12).


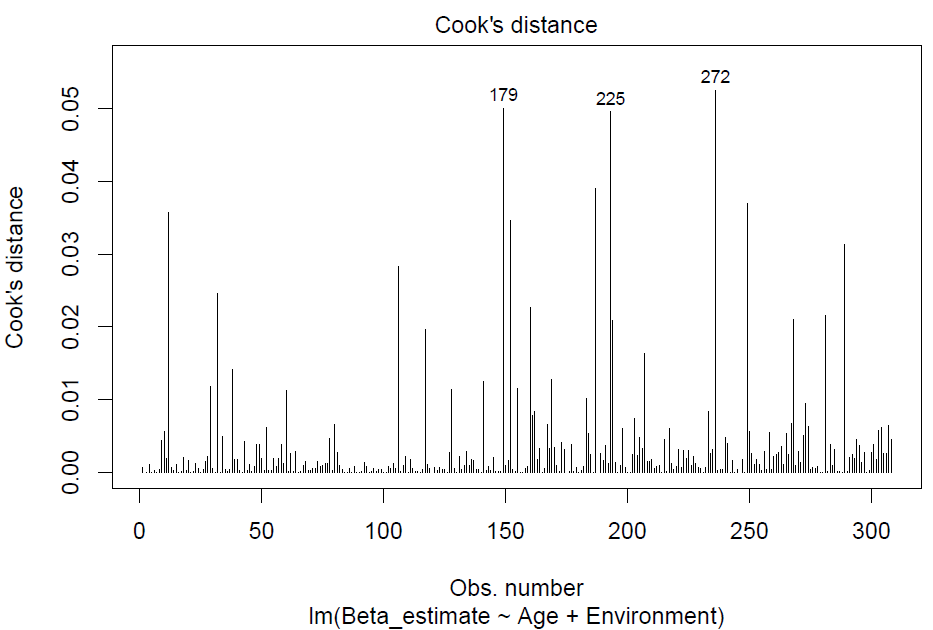


*Supplementary Figure 12:* *Plots demonstrating the absence of evidence for specific ages disproportionately affecting the association between age and participants’ stochasticity. The x-axis on these plots indicate the observations, which are higher than the number of participants as there is one observation per participant per environment. The y-axis denotes the Cook’s distance for each datapoint, with values above 0.5 indicating an influential datapoint.*

Finally, we reproduced finding of the limited evidence for an association between age and learning rates (β < 0.01, t = 1.27, *p* = .206), though there was evidence that participants utilised a lower learning rate in the volatile environment compared to the stable environment (β = 0.03, t = 2.05, *p* = .042). None of the Cook’s D statistics exceeded values of 0.08 (see Supplementary Figure 13).


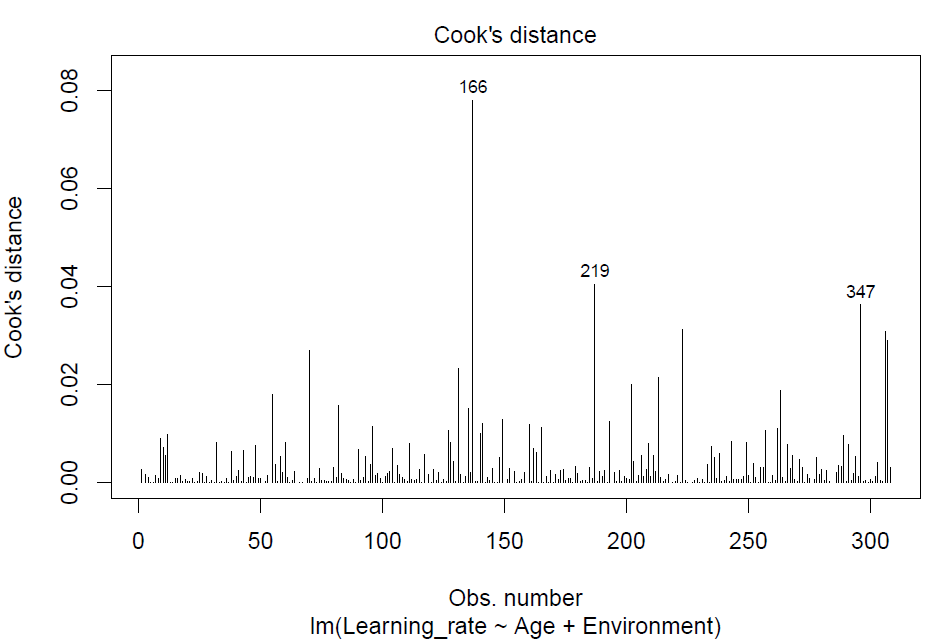


*Supplementary Figure 13: Plots demonstrating the absence of evidence for specific ages disproportionately affecting the association between age and participants’ learning rates. The x-axis on these plots indicate the observations, which are higher than the number of participants as there is one observation per participant per environment. The y-axis denotes the Cook’s distance for each datapoint, with values above 0.5 indicating an influential datapoint.*

Supplementary Reference:

Fradkin, I., & Eldar, E. (2023). Accumulating evidence for myriad alternatives: Modeling the generation of free association. *Psychological Review*, *130*(6), 1492.
